# Single-cell transcriptomics reveals a distinct developmental state of *KMT2A*-rearranged infant B-cell acute lymphoblastic leukemia

**DOI:** 10.1038/s41591-022-01720-7

**Published:** 2022-03-14

**Authors:** Eleonora Khabirova, Laura Jardine, Tim H. H. Coorens, Simone Webb, Taryn D. Treger, Justin Engelbert, Tarryn Porter, Elena Prigmore, Grace Collord, Alice Piapi, Sarah A. Teichmann, Sarah Inglott, Owen Williams, Olaf Heidenreich, Matthew D. Young, Karin Straathof, Simon Bomken, Jack Bartram, Muzlifah Haniffa, Sam Behjati

**Affiliations:** 1 Wellcome Sanger Institute, Hinxton, UK; 2 Biosciences Institute, Newcastle University, Newcastle upon Tyne, UK; 3 Haematology Department, Freeman Hospital, Newcastle-upon-Tyne Hospitals NHS Foundation Trust, Newcastle upon Tyne, UK; 4 Cambridge University Hospitals NHS Foundation Trust, Cambridge, UK; 5 Department of Paediatrics, University of Cambridge, Cambridge, UK; 6 Department of Haematology, University College London Hospital, London, UK; 7 Department of Haematology, University College London Cancer Institute, London, UK; 8 Great Ormond Street Hospital for Children NHS Foundation Trust and NIHR Great Ormond Street Hospital Biomedical Research Centre, London, UK; 9 UCL Great Ormond Street Institute of Child Health, London, UK; 10 Princess Maxima Center for Pediatric Oncology, Utrecht, the Netherlands; 11 Wolfson Childhood Cancer Research Centre, Translational and Clinical Research Institute, Newcastle University, Newcastle upon Tyne, UK; 12 The Great North Children’s Hospital, Newcastle upon Tyne Hospitals NHS Foundation Trust, Newcastle upon Tyne, UK; 13 Department of Dermatology and NIHR Newcastle Biomedical Research Centre, Newcastle upon Tyne Hospitals NHS Foundation Trust, Newcastle upon Tyne, UK

**Keywords:** Cancer genomics, Leukaemia

## Abstract

*KMT2A-*rearranged infant ALL is an aggressive childhood leukemia with poor prognosis. Here, we investigated the developmental state of *KMT2A*-rearranged infant B-cell acute lymphoblastic leukemia (B-ALL) using bulk messenger RNA (mRNA) meta-analysis and examination of single lymphoblast transcriptomes against a developing bone marrow reference. *KMT2A*-rearranged infant B-ALL was uniquely dominated by an early lymphocyte precursor (ELP) state, whereas less adverse *NUTM1*-rearranged infant ALL demonstrated signals of later developing B cells, in line with most other childhood B-ALLs. We compared infant lymphoblasts with ELP cells and revealed that the cancer harbored hybrid myeloid–lymphoid features, including nonphysiological antigen combinations potentially targetable to achieve cancer specificity. We validated surface coexpression of exemplar combinations by flow cytometry. Through analysis of shared mutations in separate leukemias from a child with infant *KMT2A*-rearranged B-ALL relapsing as AML, we established that *KMT2A* rearrangement occurred in very early development, before hematopoietic specification, emphasizing that cell of origin cannot be inferred from the transcriptional state.

## Abstract (web summary)

Single-cell transcriptomic and phylogenetic analyses reveal new insights into the developmental origin and potential therapeutic targets for a particularly aggressive form of B-cell acute lymphoblastic leukemia in infants.

## Main

Once a universally fatal disease, B-ALL of childhood is curable in the majority of cases. An exception is B-ALL arising in children younger than one year of age (infant B-ALL), which remains fatal in more than 50% of children^1,2^. Most cases (70–80%) of infant B-ALL are associated with rearrangements of the *KMT2A* gene (encoding a histone methyltransferase), which confers an especially poor prognosis^2^. Various hypotheses have been proposed to account for the aggressive nature of infant B-ALL. In particular, it has been suggested that infant lymphoblasts retain myeloid features that confer resistance to treatment strategies aimed at ALL^3^. Disappointingly, although protocols incorporating strategies from acute myeloid leukemia (AML) therapy marginally increased survival, additional intensification has not improved this further^1^. Similarly, salvage treatments that have proven successful in high-risk lymphoblastic leukemias, such as allogeneic stem cell transplantation or chimeric antigen receptor T cells targeting B-cell antigens, produce disappointing outcomes in infant B-ALL^4,5^. It is noteworthy that infant B-ALL not associated with *KMT2A* fusion, especially those with *NUTM1* gene rearrangements, confer a more favorable prognosis^6,7^ and that *KMT2A* rearrangements in the setting of adult B-ALL are also considered high risk^8^. These observations raise the question whether the aggressive clinical behavior of *KMT2A*-rearranged infant B-ALL is underpinned by a distinct cellular phenotype.

Leukemias are primarily classified by their morphological appearance or immunophenotype, as assessed by flow cytometric analyses of key hematopoietic markers and cytogenetic changes. Generally, this approach is likely to capture the differentiation state of most leukemias accurately. Occasionally, it may be erroneous when cancer cells use key hematopoietic genes aberrantly, particularly in leukemias that are driven by mutations in genes that facilitate lineage plasticity, such as *KMT2A*. In this context, a quantitative molecular assessment of hematopoietic cell states that does not rely on any individual marker, but instead builds on entire cellular transcriptomes, would provide an unbiased readout of cell states. Such high-resolution assessments are now feasible using single-cell mRNA sequencing to directly compare cancer cells to normal cells, including to fetal and adult hematopoietic cells^9–12^. We set out to study the developmental phenotype of *KMT2A*-rearranged infant B-ALL by comparing cancer cells with normal human hematopoietic cells.

## Results

### Cell signal analysis of 1,665 leukemia transcriptomes

**Fig. 1:**
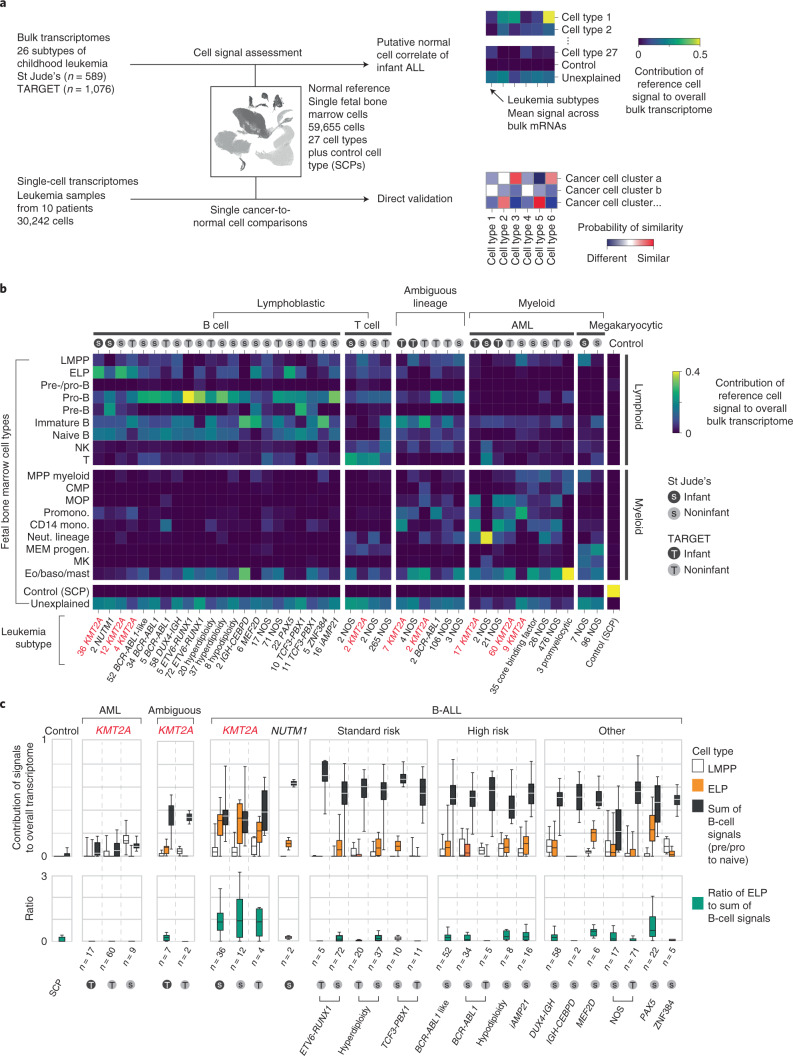
Cell signal analysis of 1,665 leukemia transcriptomes reveals an ELP state in *KMT2A*-rearranged B-ALL. **a**, Schematic overview of the study approach. We assessed the differentiation state of *KMT2A*-rearranged infant ALL by measuring signals of human fetal bone marrow cell types across the entire spectrum of childhood leukemia in data derived from two different cohorts (St Jude’s and TARGET). We then validated cell signals by single-cell mRNA sequencing for direct comparison of cancer and normal cells. **b**, Heatmap showing mean cell signals of human fetal bone marrow cells (*y* axis) in human leukemia bulk transcriptomes subdivided by genetic subtype (see labels underneath, *KMT2A* rearrangements shown in red text), age (gray circle, infant; black circle, noninfant) and source (S, St Jude’s; T, TARGET). Numbers next to labels refer to case load per subtype. Subtypes with only one case were excluded from analysis. baso, basophil; CMP, common myeloid progenitor; Eo, eosinophil; LMPP, lymphoid-primed multipotent progenitor; MEM progen., ; MK, megakaryocyte; mono., monocyte; MOP, monocyte progenitor; MPP, multipotent progenitor; Neut., neutrophil; NK, natural killer; Promono., promonocyte. **c**, Top: box and whisker plots showing proportional contribution of signals (lymphomyeloid-primed progenitor, ELP and later B-cell stages combined (i.e., pre-/pro-B, pro-B, pre-B and naive B)) to the transcriptome of leukemias (see *x* axis labels). Bottom: box and whisker plots summarizing the ratio of ELP to later B-cell stage signals. Center lines represent the median, box limits represent 25%/75% quartiles and whiskers represent minimum/maximum (top) and 1.5× interquartile range (bottom). *n* is the number of biologically independent variables, as listed below each group of plots. Risk refers to the clinical cytogenetic risk as defined in the protocol of the current European ALL trial ‘ALLTogether’ (EudraCT 2018-001795-38).

The starting point of our investigation was a meta-analysis of 1,665 bulk transcriptomes representing the entire spectrum of childhood ALL and AML across two cohorts, St. Jude Children’s Research Hospital (St Jude’s; *n* = 589) and TARGET (Therapeutically Applicable Research to Generate Effective Treatments; *n* = 1,076) (Fig. 1a and Supplementary Table 1). We determined the predominant hematopoietic cell signal within each bulk leukemia transcriptome by deconvolution. We chose a deconvolution method that uses entire transcriptomes to determine cell signals within bulk mRNA data and quantifies what proportion of the cancer bulk cannot be accounted for by normal reference cells^13^. As childhood leukemias, and infant ALL in particular, are generally thought to arise in utero^14,15^, we applied fetal hematopoietic cells as the reference in our analyses. To this end, we used recent single-cell mRNA data from ~60,000 fetal bone marrow cells, which captured the greatest breadth of fetal hematopoietic cell types to date^9^ (Supplementary Table 2). We adopted the annotation of normal cell types directly from the fetal bone marrow data analysis^9^ and supplemented the hematopoietic reference with a control fetal cell type that should not be present in human leukemia samples, Schwann cell precursors (SCPs) derived from human fetal adrenal glands^16^.

A global overview of cell signals in bulk childhood leukemia transcriptomes showed expected patterns, namely myeloid signals in myeloid leukemias, T-cell signals in T-cell ALL and imprints of the various stages of B-cell development in B-ALL (Fig. 1b and Supplementary Fig. 1). Transcriptional signatures from the control SCP population did not contribute to leukemias (negative control analysis) and matched itself (i.e., SCPs) perfectly with no unexplained signal (positive control analysis). *KMT2A*-rearranged infant B-ALL exhibited distinct cell signals with a marked contribution of ELPs. ELPs are oligopotent lymphoid precursors that are capable of differentiating along different lymphocyte lineages and that retain minimal myeloid differentiation capacity in vitro^17,18^. Defined as CD34^+^CD127^+^CD10^−^CD19^−^ cells, they sit upstream of pre-/pro-B and pro-B progenitors in the B lymphopoiesis hierarchy^18^.

### An ELP signal in *KMT2A*-rearranged B-ALL

**Extended Data Fig. 1:**
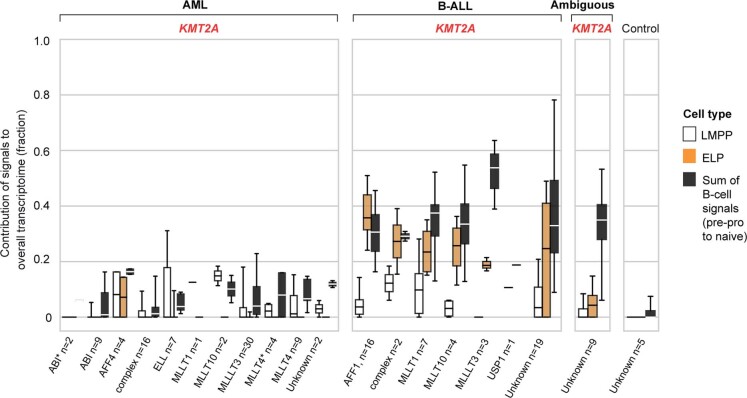
ELP signal is common to *KMT2A*-rearranged B-ALL, independent of fusion partner. Box and whisker plots showing contributions of cell signals – LMPP, ELP and latter B-cell stages (that is pre-pro-B, pro-B, pre-B and naive B combined) to the transcriptome of *KMT2A*-rearranged leukemias grouped by *KMT2A* fusion partner (see x axis labels). Centre lines=median, box limits=25%/75% quartiles, whiskers=min/max (top) and 1.5*interquartile range (bottom). n= biologically independent variables, as listed below each group of plots.

To further examine the ELP signal in *KMT2A*-driven infant B-ALL, we examined the ratio of the ELP signal over later stages of B-cell development in each leukemia subtype (Fig. 1c). This quantification demonstrated a significant shift toward the ELP state in *KMT2A*-rearranged infant ALL compared to other high (cytogenetic)-risk B-ALL subtypes (*P* < 10^−19^, Student’s two-tailed *t* test), standard (cytogenetic)-risk subtypes (*P* < 10^−31^) and currently unstratified subtypes of B-ALL (*P* < 10^−13^) (Fig. 1c). Among *KMT2A*-rearranged infant B-ALL, the ELP signal was present irrespective of fusion partners of *KMT2A* but strongest in cases harboring the most common *KMT2A* rearrangement^19^, the *KMT2A-AFF1* (*MLL-AF4*) gene fusion (*P* < 0.01 compared against other fusion partners; Mann–Whitney rank test) (Extended Data Fig. 1). The leukemias with the next highest relative ELP signals were *PAX5* and *MEF2D*-mutated B-ALL, although the ELP signals there were accompanied by stronger signals from later B-cell stages. In contrast to *KMT2A*-rearranged B-ALL, differences between ELP signals and later B-cell signals were significant in *PAX5*- and *MEF2D*-mutated B-ALL (*P* < 0.01 and *P* < 0.05, respectively; Wilcoxon signed-rank test). Although *MEF2D* mutation results in maturation arrest at the pre-B stage, its distinct immunophenotype is recognized to overlap with both early and late B progenitor stages^20^. The similarity of cell signals in *PAX5* and *KMT2A* mutant B-ALL may represent the intimate relationship of *KMT2A* and *PAX5* in regulating B lymphopoiesis^21^.

Studying the pattern of ELP signal across disease groups indicated that the signal was specific to *KMT2A* rearrangements within a B-cell context but independent of age for three reasons. First, the ELP signal was not universally associated with *KMT2A* rearrangements; neither myeloid nor ambiguous lineage leukemias with *KMT2A* rearrangements harbored appreciable ELP signals. Second, the ELP signal was not driven by young age alone, as other infant leukemias (B-ALL, ambiguous lineage leukemia and AML) exhibited no, or only minimal, ELP signal (Fig. 1c). In particular, infant B-ALL with *NUTM1* rearrangement (which carries a favorable prognosis) exhibited cell signals more reminiscent of standard-risk childhood B-ALL, with a shift away from ELPs toward later B-cell stages. Third, *KMT2A*-rearranged B-ALL of older children did exhibit marked ELP signals akin to infant *KMT2A*-driven B-ALL. Overall, these findings led us to hypothesize that, relative to other B-ALL, *KMT2A*-rearranged B-ALL exhibits a distinct hematopoietic phenotype primarily resembling ELP cells with limited signals of B-cell development.

### Direct single cancer cell to normal cell comparison

**Fig. 2:**
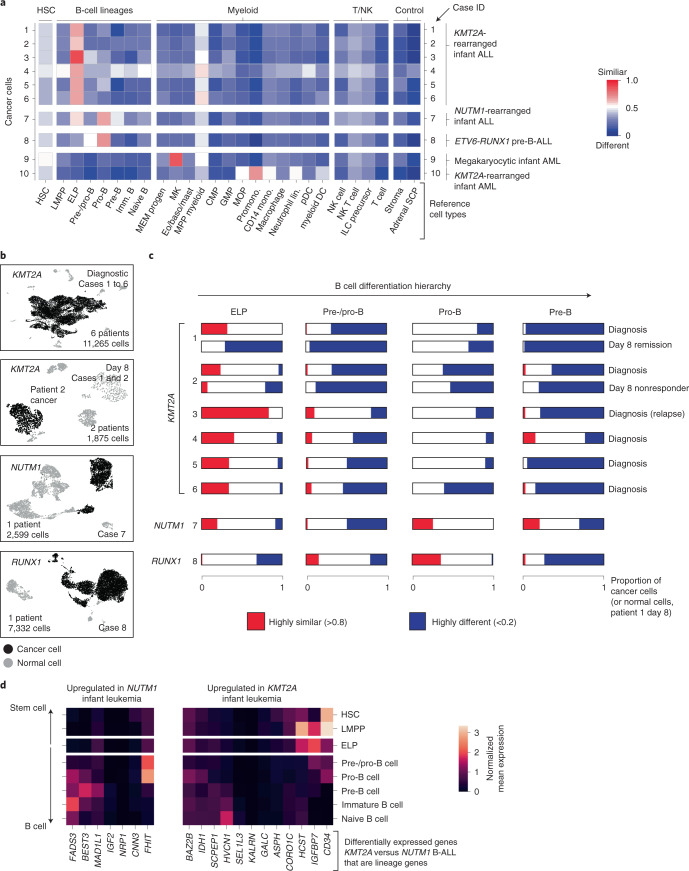
Validation of ELP signals by direct single cancer cell to normal cell comparison. **a**, Heatmap comparing cell clusters from diagnostic specimens (*y* axis) to normal human fetal bone marrow cell types (*x* axis; bold labels highlight cell types shown in C). Cell clusters represent cancer (as defined by clinical diagnostic flow cytometric profiles, see Extended Data Fig. 2) and normal cells of individual patient samples (as per case ID number; see Supplementary Table 3 for an overview of patients). All are diagnostic samples at presentation, except case 3 (relapse presentation). Heat colors represent the mean probability (across the cell cluster) of a match as determined by logistic regression (red, similar; blue, different). DC, dendritic cell; GMP, granulocyte–monocyte progenitor; HSC, hematopoietic stem cell; ILC, innate lymphoid cell; Imm., immature; lin., lineage; pDC, plasmacytoid dendritic cell. **b**, Uniform manifold approximation and projection of B-ALL scRNA-seq data divided by genetic subtype. *KMT2A-*rearranged B-ALL at diagnosis and day 8 of treatment are presented separately. Within each heatmap, black dots represent cancer and gray dots noncancer. **c**, Per cancer cell (normal cells for day 8 remission samples of patient 1) logistic regression score against reference B-lineage cell states, with thresholds of >0.8 indicating similarity (red) and <0.2 indicating dissimilarity (blue). **d**, Subset of differentially expressed genes between infant *KMT2A*-rearranged B-ALL and *NUTM1*-rearranged B-ALL. *x* axis, gene name; *y* axis, fetal bone marrow cell type. Heatmap shows the average gene expression per reference cell type for genes up-regulated in *NUTM1* B-ALL (left) and *KMT2A* B-ALL (right).

**Extended Data Fig. 2:**
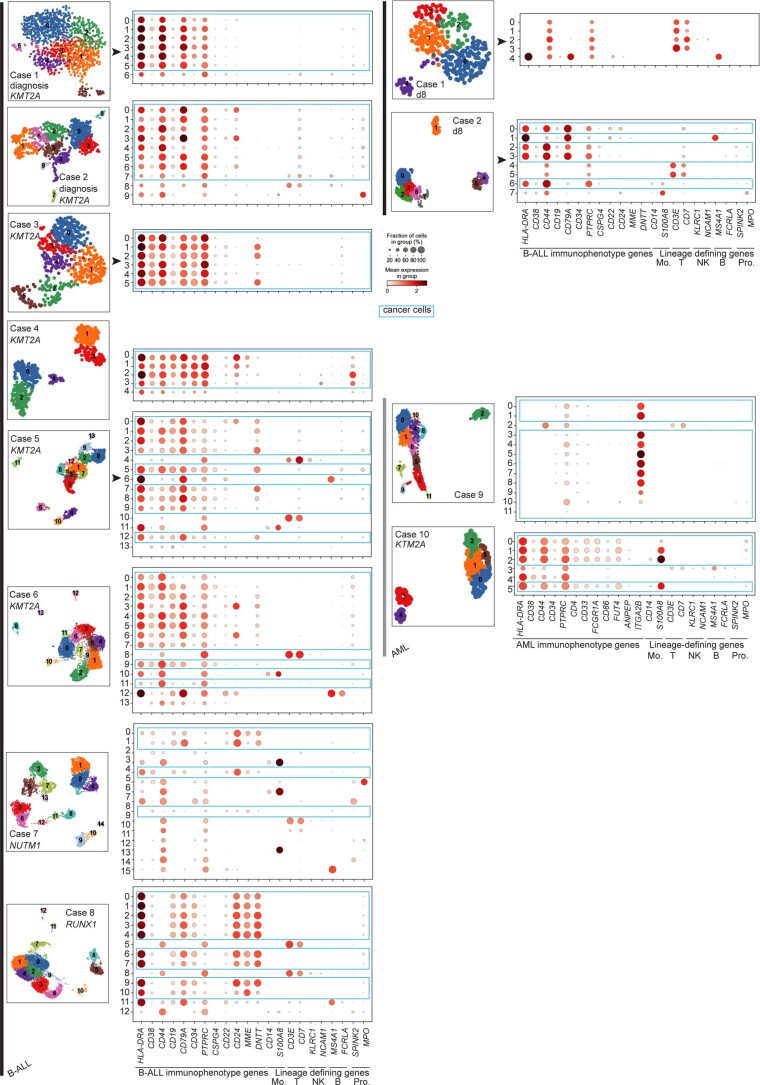
Identification of cancer cells in leukemia scRNA-seq data using immunophenotype gene expression. UMAP projections of leukaemia scRNA-seq data sets, coloured by Louvain cluster. Accompanying dotplots show per-cluster expression of B-ALL immunophenotype genes or AML immunophenotype genes and lineage-defining genes of monocytes (Mo.), T cells (T), NK cells (NK), B cells (B) and progenitors (Pro.). Dot colour denotes log-transformed, normalised and scaled gene expression value, while dot size indicates percentage of cells in each cluster expressing the stated gene. Immunophenotypes are provided in Supplementary Table 4.

To validate and further explore this proposition, we performed single-cell RNA-sequencing (scRNA-seq) analysis (10x Genomics) of diagnostic specimens from six infants with *KMT2A*-rearranged infant B-ALL, including a relapse presentation (case 3) and additional day 8 specimens from responding (case 1) and nonresponding (case 2) patients. We compared these to four other leukemias: *NUTM1*-rearranged infant B-ALL (*n* = 1), *KMT2A*-rearranged infant AML (*n* = 1), megakaryoblastic neonatal AML (*n* = 1) and childhood *ETV6-RUNX1* B-ALL (a common subtype of standard-risk childhood B-ALL; *n* = 1) (Supplementary Table 3). From these 12 diagnostic leukemia samples, we obtained a total of 30,242 cells, including 23,286 cancer cells that we identified based on gene expression matching patient-specific diagnostic flow cytometric profiles (Supplementary Table 4 and Extended Data Fig. 2). Using a published cell-matching method based on logistic regression^12,16^, we directly compared leukemia transcriptomes with mRNA profiles of human fetal bone marrow cells to determine which normal cell type the cancer cells most closely matched. We found that *KMT2A*-rearranged infant B lymphoblasts overwhelmingly resembled ELP cells at diagnosis and relapse and in nonresponding disease (Fig. 2a–c). By contrast, non-ELP cell signals predominated in other types of leukemia, precisely as predicted from the initial deconvolution analysis (Fig. 1b). In particular, in the aforementioned subtype of infant B-ALL with a favorable prognosis, *NUTM1*-rearranged infant B-ALL, single-cell analysis confirmed the shift toward pre-B-cell states and away from ELPs. To further explore the differences between *KMT2A*- and *NUTM1*-driven infant B-ALL, we performed independent differential gene expression analysis of bulk transcriptomes and single-cell data, which yielded an overlapping list of 90 differentially expressed genes (Methods). Focusing on genes used in normal fetal bone marrow, we found that in *KMT2A* B-ALL, genes of early B-cell development were overexpressed, whereas in *NUTM1* B-ALL genes of more differentiated B cells predominated (Fig. 2d and Supplementary Table 5). These findings thus corroborate our proposition that the differentiation state of *NUTM1* blasts, similar to *ETV6-RUNX1* blasts, is shifted toward later stages of B-cell development.

**Extended Data Fig. 3:**
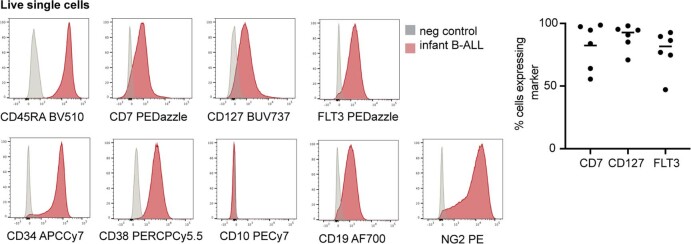
Immunophenotype of *KMT2A*-rearranged infant B-ALL. Left: Histograms showing surface antigen expression in a representative primary *KMT2A-*rearranged infant B-ALL sample relative to negative control. Right: Scatterplot showing percentage of cells in each sample expressing ELP-characteristic antigens > negative control (n = 2 xenograft, n = 4 primary *KMT2A*-rearranged infant B-ALL). Line= mean; 82% CD7^+^, 93% CD127^+^ and 82% FLT3^+^).

To determine the heterogeneity of B-cell states within patients, we performed logistic regression on a per-cell basis (Fig. 2c). This revealed in every case of *KMT2A-*rearranged ALL that the greatest proportion of blasts with a close match to a specific developing B-cell type resembled ELP cells. Similarly, very few infant *KMT2A* lymphoblasts were dissimilar to ELP cells. By contrast, the developmental phenotype of *NUTM1* and *ETV6*-*RUNX1* lymphoblasts was shifted toward later B-cell stages, peaking at the pro-B-cell stage in terms of the similarity and dissimilarity of individuals blasts to fetal cells. Finally, we assessed by flow cytometry a set of six *KMT2A* infant B-ALL samples, including four primary samples (three independent of the single-cell scRNA-seq cohort) and two xenografts derived from these patients. We demonstrated an ELP-like immunophenotype in 80–90% of cells (Extended Data Fig. 3). Together, these findings confirm that an ELP-like developmental state predominates in *KMT2A* infant B-ALL at diagnosis and relapse in resistance and after xenotransplantation.

### Phylogenetic timing of the origin of infant ALL

**Fig. 3:**
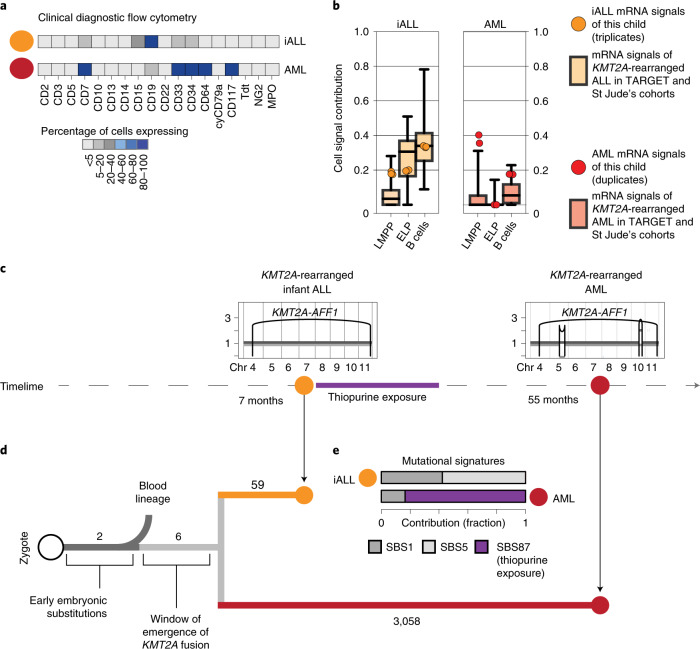
Phylogenetic timing of the origin of infant ALL. **a**, Diagnostic flow cytometry of two leukemias that arose in the same child 4 years apart: *KMT2A*-rearranged infant ALL (yellow, abbreviated iALL) and *KMT2A-*rearranged AML (red). MPO, myeloperoxidase. **b**, Cell signal assessment of bulk transcriptomes generated from this child (ALL in technical triplicates, AML in technical duplicates) shows that the cell signals (LMMP, ELP and B cells (i.e., the sum of all B-cell signals)) of ALL (yellow circle) and AML (red circle) follow the pattern of *KMT2A*-rearranged ALL (left, boxplots in background, *n* = 52 biologically independent samples) and *KMT2A*-rearranged AML (right, boxplots in background, *n* = 86 biologically independent samples), as defined in the St Jude’s and TARGET cohorts. Boxplot center line represents the median, whiskers represent minimum/maximum and box limits represent 25%/75% quartiles. **c**, Timeline with copy-number profiles of chromosomes 4 to 11 (all other chromosomes were diploid) in both leukemias showing chromosomes (*x* axis) and copy number (*y* axis), alleles (light and dark grey lines) and rearrangement breakpoints (black vertical lines and arcs), including the chromosome 4:11 translocation underpinning the *KMT2A-AFF1* fusion. **d**, Phylogeny of blood and leukemia lineages with substitution burden defining each branch (number). **e**, Assessment of mutational signatures as defined by the trinucleotide context of substitutions (nomenclature as per Alexandrov et al.^22^) highlighting in purple the dominant contribution (as percentage of all clonal substitutions) of signature 87 to AML. This signature is thought to be due to thiopurine agents that the child had received for ALL treatment (see timeline).

A key question raised by our findings is whether ELPs are the cells of origin of *KMT2A*-rearranged infant B-ALL or whether leukemia cells arise from another precursor and differentiate/dedifferentiate into an ELP-like state at which they arrest. A rare case of lineage switching from *KMT2A*-rearranged infant B-ALL to *KMT2A*-rearranged AML provided the opportunity to directly determine the cell of origin in phylogenetic temporal terms (Fig. 3a). We first assessed cell signals in bulk transcriptomes (in replicates) derived from a child with *KMT2A*-rearranged B-ALL and AML. Once again, we observed that ALL, but not AML transcriptomes, exhibited an ELP signal (Fig. 3b). To determine the phylogeny of the cancers, we performed whole-genome DNA sequencing of AML, ALL and remission bone marrow and called all classes of variants using an extensively validated mutation-calling pipeline^22^ (variant list in Supplementary Table 6). We determined the phylogeny of each leukemia and remission bone marrow. The remission sample and leukemias shared two mosaic (early embryonic) base substitutions, representing the first cell divisions of the zygote^23,24^. Thereafter, normal blood and leukemia lineages diverged. The common leukemia lineage (that is mutations shared between ALL and AML, but not the remission sample) composed only six base substitutions along with the *KMT2A* rearrangement (Fig. 3c,d), defining an early developmental window during which the translocation formed. Assuming a mutation rate of at least 0.9 substitutions per cell division, as recently established in human fetal hematopoietic cells^25^, six substitutions would place the emergence of the *KMT2A* rearrangement in early embryonic development, before hematopoietic cell specification. After acquisition of the *KMT2A* fusion, the leukemia lineages diverged and gave rise to independent cancers, each exhibiting distinct phenotypes and somatic changes (including point mutations, copy-number profiles and mutational signatures) (Fig. 3c–e). Although this single case may not be representative of infant ALL generally or lineage-switch leukemias specifically, it demonstrates that the transcriptional state of cancer cells cannot unambiguously be used to infer its cell of origin.

### Therapeutic hypotheses based on the ELP state of infant ALL

**Fig. 4:**
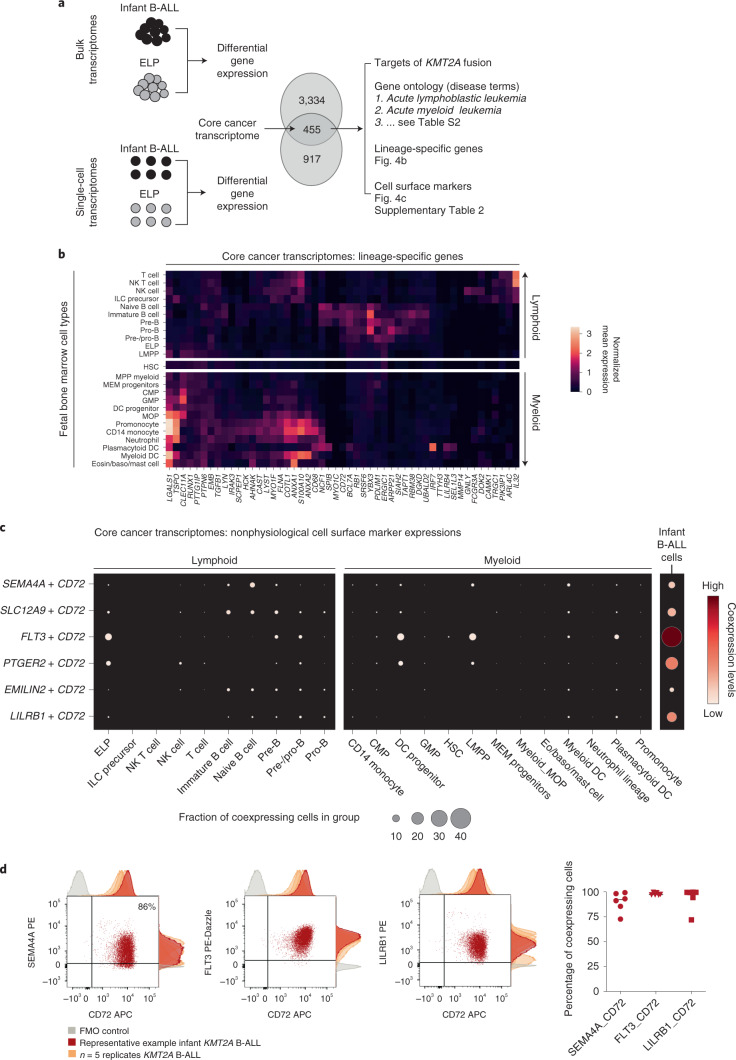
Therapeutic hypotheses based on the ELP state of infant ALL. **a**, Distilling the core cancer transcriptome (i.e., differential gene expression between infant ALL and ELP cells from bulk and single-cell data) to generate a cross-validated gene list that we annotated in three ways (right). **b**, The core cancer transcriptome encodes a mixed myeloid–lymphoid phenotype. Shown is the log normalized expression (heat color) of genes (*x* axis) that have relative lineage specificity in normal fetal bone marrow cell types (*y* axis). Eosin, eosinophil. **c**, Examples of nonphysiological combinations of cell surface markers that the core cancer transcriptome encompasses. *x* axis, fetal bone marrow cell type or infant B-ALL lymphoblasts; *y* axis, marker combinations. Dots represent coexpression of the markers (average of the product of gene expression). Dot size represents the percentage of cells in the cluster that express both markers, and heat color represents the normalized coexpression level. **d**, Left: dotplots showing coexpression of antigen combinations in a representative primary *KMT2A*-rearranged infant B-ALL sample, as measured by flow cytometry on live, single CD34^+^CD19^+^ blasts. Adjunct histograms show fluorescence-minus-one (FMO) negative controls (gray) and antigen expression in the representative sample (red) compared with *n* = 2 xenograft samples and *n* = 3 further primary infant B-ALL samples (orange). APC, allophycocyanin; PE, phycoerythrin. Right: scatterplot demonstrating the percentage of cells in each sample with expression of antigen pair higher than the fluorescence-minus-one control (line represents median).

To distill the oncogenic features of the *KMT2A*-rearranged infant B-ALL transcriptome, we directly compared leukemia with ELP transcriptomes. We determined in independent analyses the differential gene expression between bulk *KMT2A*-rearranged infant B-ALL and published bulk ELP transcriptomes^18^ and between single lymphoblast and single ELP cell transcriptomes (Fig. 4a). The overlap of these two independent analyses (Supplementary Table 7, *N* = 455) provided a cross-validated gene set, hereafter referred to as the cancer core transcriptome, that differentiates *KMT2A-*rearranged B lymphoblasts from their closest normal cell correlate (i.e., ELPs), which we annotated in five ways. First, we queried whether the cancer core transcriptome contained known target genes of the *KMT2A-AFF1* fusion^26^, the most common *KMT2A* rearrangement in B-ALL. We found 63 of 455 genes to be targets of the *KMT2A-AFF1* fusion, which represents a significant enrichment (*P* < 10^−107^, as assessed in a Monte Carlo simulation; Methods and Supplementary Table 7). Second, we discerned the lineage-independent effects of *KMT2A* translocation by overlapping the *KTM2A-*rearranged B-ALL cancer core transcriptome with genes differentially expressed in *KMT2A*-driven AML (relative to its normal cell correlate, monocyte progenitors (MOPs); case 10, Fig. 2a). We identified an overlapping gene set of 67 genes that, according to gene ontology annotations, disrupted key regulatory processes such as cell communication, proliferation and development and promoted expression of genes maintaining a primitive state (*HOXA6, BMI1* and *MEIS1*) (Supplementary Tables 7 and 8). Third, we asked whether the cancer core transcriptome encompassed lineage-specific genes by interrogating normal fetal bone marrow cells. We found that a subset of genes (*n* = 51) was lineage specific, representing either lymphoid or myeloid cell types (Fig. 4b). Fourthly, we annotated the cancer core transcriptome by gene ontology analysis. The top two disease annotations were lymphoblastic and myeloid leukemia, further suggesting that the cancer core transcriptome encoded a hybrid myeloid–lymphoid phenotype (Supplementary Table 7). Finally, we identified cell surface antigens among differentially expressed genes, as many novel treatments in childhood leukemias center on targeting blast markers through antibodies or genetically modified T cells. A total of 41 of 455 genes encoded surface markers, some of which were relatively specific to myeloid (*n* = 18) or lymphoid (*n* = 4) lineages, generating 72 potential nonphysiological marker combinations (Supplementary Table 7). Examples of nonphysiological coexpression patterns that were particularly specific to infant B-ALL are shown in Fig. 4c. Interestingly, these were centered on the lymphoid marker CD72, which a proteomic screen recently implicated as a target in infant ALL^27^. Coexpression of nonphysiological combinations was measured by flow cytometry, where commercial antibodies existed, confirming that dual-targeting would encompass >90% of leukemic cells (Fig. 4d). Now that surface marker therapies targeting two antigens simultaneously are already in use, nonphysiological coexpression of markers may represent an attractive therapeutic avenue in infant B-ALL.

## Discussion

In clinical diagnostic and therapeutic terms, *KMT2A*-rearranged infant B-ALL is considered to be a B-precursor leukemia. Based on independent data and analytical techniques, we arrived at the conclusion that infant lymphoblasts most closely resemble human fetal ELPs. This ELP-like transcriptional phenotype distinguishes *KMT2A*-rearranged infant B-ALL from other childhood B-ALLs.

A key question that our findings raise is whether the ELP-like state accounts for the poor prognosis of *KMT2A*-rearranged infant B-ALL. Three observations lend credence to this proposition. First, in both bulk mRNA and single-cell analyses, *NUTM1*-rearranged infant B-ALL, recently identified to carry a favorable prognosis^6^, exhibited cell signals away from the ELP state and more reminiscent of standard-risk B-ALL. Second, we observed an ELP-like state in older children with B-ALL *KMT2A* rearrangements, in whom *KMT2A* fusions are considered a high-risk cytogenetic change that mandates treatment intensification^28^. Third, B-ALLs with the next highest relative ELP signals (*PAX5* alterations and *MEF2D* mutations) are also considered high risk^20,29^. These observations raise the possibility that ELP features confer a high-risk clinical phenotype in B-ALL while recognizing the challenge of separating this signal from the prognostic significance of cytogenetic changes.

Considerable efforts to identify the cell of origin in leukemias have arisen from the promise that targeted clearance will result in durable remission. Focusing in on the cell of origin in *KMT2A*-rearranged infant B-ALL, key pieces of evidence are (1) rearrangement is prenatal event, as demonstrated by Guthrie card examinations and concordance in monozygotic twins^14^; (2) rearrangement in the hematopoietic compartment is observed in CD34^+^CD19^−^ cells^30^, before VDJ recombination in most cases, resulting in low frequency of clonal immunoglobulin rearrangements^31,32^; and (3) rearrangement may also be seen in bone marrow mesenchymal cells, suggesting a prehematopoietic origin in some^33^. We directly determined the phylogenetic origin of an infant leukemia in a rare case of a child in whom infant B-ALL and childhood AML developed, both harboring *KMT2A* rearrangements. The number of shared mutations between these leukemias suggests that the *KMT2A* rearrangement arose before gastrulation and specification of hematopoiesis. With the important caveat that this case will not represent all *KMT2A-*rearranged B-ALL, it demonstrates that the cell of origin cannot be inferred from the transcriptional phenotype of leukemia cells. Although our results demonstrate the consistency of an ELP transcriptional state in *KMT2A* B-ALL cells at diagnosis, in resistant disease, at relapse and in xenografts, further studies are required to establish whether an ELP signal can be traced back to disease-initiating cells.

The benefit of accurately defining the transcriptional state of *KMT2A-*rearranged infant B-ALL is the ability to devise novel strategies for targeted therapy. We compared leukemic blasts with fetal bone marrow ELPs from independent data sets to yield a core cancer transcriptome, which was characterized by fusion gene targets and a mixture of lymphoid and myeloid lineage genes. We identified nonphysiological combinations of surface antigen genes and demonstrated that these combinations are coexpressed as surface proteins, potentially allowing >90% of leukemic blasts to be destroyed by dual-targeting tandem-chimeric antigen receptor T-cell or bispecific antibody therapies. Targeting combinations of antigens from different lineages simultaneously may afford exquisite specificity for cancer cells.

The quantitative molecular approach we deployed here, leveraging large archives of bulk mRNAs, emerging reference catalogs of normal human cells and direct examination of single blast transcriptomes, lends itself for reappraising the phenotype of human leukemias to derive novel biological and therapeutic hypotheses. As leukemias are primarily classified by their hematopoietic phenotype, we propose that *KMT2A*-rearranged infant B-ALL be considered an ELP-like leukemia.

## Methods

### Ethics statement

Patient blood and bone marrow samples were obtained from the Newcastle Biobank (as approved by Newcastle and North Tyneside 1 Research Ethics Committee, reference 17/NE/0361) or Great Ormond Street Hospital for Children diagnostic archives (as approved by the National Research Ethics Service Committee London Brent, reference 16/LO/0960). Informed consent was obtained from all participants. Patient-derived xenograft (PDX) samples were generated in accordance with the UK Animals (Scientific Procedures) Act 1986 under project licenses PPL60/4552 and PPL60/4222 following institutional ethical review.

### Sample preparation

Peripheral blood mononuclear cells were prepared from blood, bone marrow or PDX samples by density centrifugation using Lymphoprep (Stemcell) according to manufacturer’s instructions. Samples were cryopreserved in fetal bovine serum (FBS) with 10% dimethyl sulfoxide and stored in liquid nitrogen. PDXs were generated by intrafemoral transplant (under isoflurane anesthesia) of 10^6^ patient blood or bone marrow cells into NOD.Cg-Prkdcscid Il2rgtm1Wjl/SzJ mice (Charles River Laboratories and bred in-house) aged 8–10 weeks old^34^. PDX cells were harvested from engrafted bone marrow or spleen.

### Flow cytometry

Cryopreserved ALL samples (*n* = 4 primary samples, *n* = 2 PDXs; Supplementary Table 3) were thawed in RF-10 (RPMI, Sigma-Aldrich) supplemented with 10% (v/v) heat-inactivated FBS (Gibco), 100 U ml^−1^ penicillin (Sigma-Aldrich), 0.1 mg ml^−1^ streptomycin (Sigma-Aldrich) and 2 mM l-glutamine (Sigma-Aldrich). Up to one million cells were stained with antibody cocktail, incubated for 30 min on ice, washed with flow buffer (PBS containing 5% (v/v) FBS and 2 mM EDTA), and resuspended in flow buffer with DAPI (Sigma-Aldrich) added to a final concentration of 3 μM. Antibodies for immunophenotyping (Extended Data Fig. 3) were (clone, supplier) NG2 PE (9.2.27, BD Biosciences), FLT3 PEDazzle (BV10A4H2, Biolegend), CD10 PECy7 (HI10a, Biolegend), CD2 fluorescein isothiocyanate (FITC) (S5.2, BD Biosciences), CD3 FITC (SK7, BD Biosciences), CD14 FITC (MφP9, BD Biosciences), CD16 FITC (NKP15, BD Biosciences), CD56 FITC (NCAM16.2, BD Biosciences), CD235a FITC (GA-R2, BD Biosciences), CD38 PERCPCy5.5 (HB-7, Biolegend), CD45RA BV510 (HI100, BD Biosciences), CD7 BV650 M-T701, BD Biosciences), CD127 BUV737 (HIL-7R-M21, BD Biosciences), CD90 APC (5E10, Biolegend), CD19 AF700 (HIB19, Biolegend) and CD34 APCCy7 (581, Biolegend). Antibodies for nonphysiological antigen coexpression profiling (Fig. 4) were SEMA4A PE (T9-10, BD Biosciences) or LILRB1 PE (GHI/75, Biolegend); FLT3 PE Dazzle (as above); CD10 PECy7 (as above); CD19 FITC (4G7, BD Biosciences), ICOSLG BV510 (2D3/B7-H2l, BD Biosciences), CD127 BUV737 (as above), CD72 APC (SF3, Biolegend) and CD34 APCCy7 (as above). All antibodies were used at 1:25 dilution, except for CD10 PECy7 and CD127 BV650, which were used at 1:50 dilution. For fluorescence-minus-one controls, cells and antibody cocktails were prepared identically but without the antibody of interest. FACS was performed on a BD FACSAria running DIVA v.8, and data were analyzed using FlowJo (v.10.6.2, BD Biosciences). Thresholds for negative expression were set using fluorescence-minus-one controls for Fig. 4 analysis and using negative cells in PDX samples (mouse splenocytes) for Extended Data Fig. 3 analysis.

### Bulk RNA sequencing

Total RNA from the lineage-switch case (Fig. 3) was extracted from peripheral blood mononuclear cells using RNeasy Mini Kit (Qiagen, 74106) and mRNA captured using NEBNext Ultra Directional RNA Kit with NEBNext poly(A) mRNA Magnetic Isolation Module. Paired-end 150-bp sequencing was performed on HiSeq 4000 (Illumina), with transcript abundance quantified from raw reads via Salmon v.0.8.2 and alignment performed against the hg38 reference human transcriptome (GENCODE release 27).

### scRNA-seq

Thawed cells were manually counted and 7,000 cells added to each channel of a Single Cell Chip before loading onto the 10x Chromium Controller (10x Genomics). Reverse transcription, cDNA amplification and sequencing libraries were generated using the Single Cell 3’v2 (P1_iALL, P2_iALL, P9_iAML), Single Cell 3’v3 (P3_iALL, P4_iALL, P10_iAML) and Single Cell 5’ v1 (P5_iALL, P6_iALL, P7_iALL_NUTM1 and P8_iALL_ETV6) Reagent kit (10x Genomics) according to the manufacturer’s instructions. Libraries were sequenced using an Illumina HiSeq 4000 with v.4 SBS chemistry. All libraries were sequenced to achieve a minimum of 50,000 reads per cell.

### Alignment, quantification and quality control

Raw fastq files for scRNA-seq data for P1_iALL,P2_iALL, P9_iAML (single-cell 3’ v2 kit) were processed with the Cell Ranger v2.0.2 (ref. ^35^) pipeline, and the rest of the samples were processed at a later time point with the Cell Ranger v3.0.2 pipeline, which aligned the reads to the reference human genome (GRCh38 v1.2.0) and produced a matrix of gene expression per cell. Ambient mRNA contamination was removed with SoupX package v1.4.8 in R with default parameters. Demultiplexing of P1_iALL/P2_iALL, P3_iALL/P10_iAML and P5_iALL/InfALL_classSwitch was performed with souporcell package v2.0 (ref. ^36^) with default parameters and setting number of clusters -k to 2 and–min_ref to 4 --min_alt to 4. Souporcell inferred the cluster assignment (either 0 or 1) for each cell, and given gender information (Supplementary Table 3), we were able to demultiplex the data by checking the sex-specific gene expression in each souporcell cluster (XIST for female and RPS4Y1, ZFY and a couple of others for male). Resulting gene expression matrices were further processed in python with scanpy package v1.4.4.post1 (ref. ^37^), and single cells were filtered to retain cells expressing >200 genes and having mitochondrial content <20%. The code used for demultiplexing and filtering is included as a Jupyter Notebook in the Code availability section.

### Dimensional reduction, clustering and annotation

After filtering for low-quality genes, single-cell data were processed in a scanpy package in python, and the total number of counts per cell were normalized to 10.000 in order to correct for library size differences; normalized data were further log-transformed. Principal-component analysis was performed on log-transformed data using default parameters (*N* = 50), followed by computation of neighborhood graph with default parameters (*N* neighbors = 15) and embedding the graph in two dimensions using uniform manifold approximation and projection. Clustering of single-cell data has been performed by Louvain community detection on neighborhood graph with default resolution set to 1. Clusters were assigned as cancer or noncancer, based on expression of B-ALL or AML immunophenotype genes (derived from expression profiles in clinical diagnostic panels and lineage-defining genes of monocytes, B cells, T cells, natural killer cells or progenitors; Extended Data Fig. 2 and Supplementary Table 4).

### Logistic regression analysis

To test the probability that cancer cell transcriptomes are similar normal reference transcriptomes (single-cell fetal bone marrow dataset), we used logistic regression as described previously^12,16^. Briefly, a logistic regression model was trained in R using cv.glmnet function on a fetal bone marrow dataset combined with SCP single cells from the fetal adrenal reference map^16^, setting the elastic mixing parameter alpha to 0.99, thus ensuring strong regularization. This model was then used to predict the probabilistic score of similarity of single cells in infant leukemia dataset to cell type in the fetal bone marrow dataset.

### Published bulk RNA-sequencing data

Pediatric tumor bulk RNA-sequencing data for childhood leukemia was obtained from the St. Jude Cloud and TARGET, together with associated metadata. Bulk RNA-sequencing data of human fetal bone marrow ELPs^18^ were extracted from the Gene Expression Omnibus with accession number GSE122982. Data were quantified and mapped with Salmon v. 0.13.1 (ref. ^38^) with default parameters, and transcript-level estimates were summarized with tximport package v1.14.2 in R.

### Deconvolution of bulk RNA-sequencing data

The fetal BM scRNA-seq dataset was used as a reference to infer the cell type composition in bulk RNA-sequencing data using a previously published method of deconvolution called cellular signal analysis^13^. Briefly, this method aims to predict the contribution of the normal mRNA signal to each of the bulk transcriptomes. The advantage of using cellular signal analysis over other deconvolution methods is the reporting of the ‘unexplained signal’ when the bulk transcriptome differs from all the signals in the normal reference dataset and represented as an ‘Intercept’ term. The model fit is based on tensorflow framework v1.14.0 and was run specifying gene weights using the geneWeights.tsv file that was supplied with the package and using default parameters for other arguments.

### Differential gene expression analysis

Differential gene expression analysis was performed using DESeq2 package v1.26.0 (ref. ^39^) in R. For bulk RNA-sequencing data (childhood leukemia data and ELP data) a DESeq dataset was constructed from tximport object (from Salmon quant.sf files for both childhood leukemia and ELP and creating metadata table with ‘group’ column variables set to either ‘cancer’ or ‘ELP’). For the single-cell leukemia dataset, pseudobulk was created from single cells by summarizing counts for each patient. For the single-cell ELP, MOP or NUTM1 dataset, a matrix of counts was imported in Seurat and data were subsequently clustered using default parameters. Pseudobulk was created for each ELP cluster (five in total), MOP cluster (four in total) and NUTM1 cluster (eight in total) by summarizing raw counts. Standard differential expression analysis was run using the DESeq function, and the result was filtered to only include genes with adjusted *P* value less than 0.05 and log_2_ fold changes greater than 1.

### Gene ontology analysis

Gene ontology analysis was performed using WebGestalt (WEB-based Gene SeT AnaLysis Toolkit)^40^. The gene list was defined as the overlap of differentially expressed genes between bulk *KMT2A*-rearranged infant B-ALL and bulk ELP transcriptomes and between single lymphoblast and single ELP cell transcriptomes (*N* = 455). Overrepresentation analysis was run using the human genome as a reference gene set and setting the disease phenotype database OMIM as a functional database.

### Analysis of enrichment of *KMT2A-AFF1* targets

Gene targets for the *KMT2A-AFF1* fusion (*N* = 1,052) were extracted from Kerry et al.^26^. Enrichment of these 1,052 gene targets within the core leukemia transcriptome (*N* = 455) was assessed using a Monte Carlo approach by randomly drawing 455 genes from the possible transcriptome of 33,660 genes. This step of randomly drawing the list of genes was repeated 1,000 times, and *P* values were estimated by Student’s *t* test.

### DNA sequencing and variant calling (lineage-switch case)

#### DNA sequencing and alignment

Short-insert (500-bp) genomic libraries were constructed, and 150-bp paired-end sequencing clusters were generated on the Illumina HiSeq XTen platform using no-PCR library protocols. DNA sequences were aligned to the GRCh37d5 reference genome by the Burrows–Wheeler algorithm (BWA-MEM v0.7.16a)^41^.

#### Variant calling

All classes variants were called using the extensively validated pipeline of the Wellcome Sanger Institute, built on the following algorithms: CaVEMan v1.13.14 (ref. ^42^) for base substitutions, PINDEL v2.2.4 for insertions/deletions^43^, ASCAT v4.0.1 (ref. ^44^) and Battenberg v3.2.2 (ref. ^45^) for copy-number changes and BRASS v6.0.5 for structural variants^46^.

#### Phylogenetic analyses from substitutions

We applied a previously developed framework^47–49^. In brief, beyond the standard postprocessing flags used in CaVEMan, we filtered out substitutions affected by mapping artefacts by setting the median alignment score of reads supporting a mutation ≥140 and requiring that fewer than half of the reads were clipped (CLPM = 0, CLPM, median number of soft clipped bases in variant supporting reads). Across all samples from PD38257, we recounted substitutions that were called in either blood or tumor from the patient using a cutoff for read mapping quality (28) and base quality (25). Germline variants were removed using one-sided exact binomial test on the number of variant reads and depth present (in diploid samples) to test whether the observed counts were consistent with a true variant allele frequency of 0.5 (or 0.95 for XY chromosomes). Resulting *P* values were corrected for multiple testing using the Benjamini-Hochberg method and a cutoff was set at *q* < 10^-−5^. Variants were also filtered out if they were called in a region of consistently low or high depth in diploid regions. Variants were kept if their corresponding site had a mean depth of between 20 and 60 for autosomes and a mean depth of between 10 and 30 for the X and Y chromosome. Using a beta-binomial model of site-specific error rates as previously described^47–49^, we distinguished true presence of somatic variants from support due to noise. All shared substitutions were further visually inspected in the genome browser Jbrowse^50^. The final list substitutions included in our analyses can be found in Supplementary Table 6.

#### Classification of single-nucleotide variants

To distinguish subclonal from clonal mutations in the tumor samples, we used a binomial mixture model to deconvolve the mutation counts into separate components. For each component, the optimal binomial probability and mixing proportion was estimated using an expectation-maximization algorithm. The optimal number of components was determined by the Bayesian information criterion. If the binomial probability of a component approximated the expected variant allele frequency (0.5 for diploid regions) adjusted for tumor purity, then the mutations assigned to that cluster were classified as clonal. If the estimated binomial probability for a component was lower, it was classified as subclonal.

#### Mutational signature analysis

Mutation signatures were fitted to the trinucleotide counts of single-nucleotide variants in the main clone and subclone of ALL (PD38257a) and AML (PD38257c) using the SigFit algorithm^51^ and the COSMIC reference database of mutational signatures (https://cancer.sanger.ac.uk/signatures/sbs/, v3.2), as used previously^52^. Initially, all reference signatures were fitted to the mutation counts. Only signatures that contributed at least 2% were retained during the subsequent fitting. Where mutation counts were low (<100), erroneous C>T signatures, such as those from ultraviolet light exposure (SBS7a) or mismatch repair deficiency (SBS6), were attributed to the samples. Because of their biological implausibility, these signatures were removed from the final set of fitted signatures.

### Reporting Summary

Further information on research design is available in the Nature Research Reporting Summary linked to this article.

## Online content

Any methods, additional references, Nature Research reporting summaries, source data, extended data, supplementary information, acknowledgements, peer review information; details of author contributions and competing interests; and statements of data and code availability are available at 10.1038/s41591-022-01720-7.

## Supplementary information

Supplementary Information Supplementary Figure 1 and legends for supplementary tables.

Reporting Summary

Supplementary Table 1 Supplementary Tables 1–8.

## Figures and Tables

**Extended Data Fig. 4:**
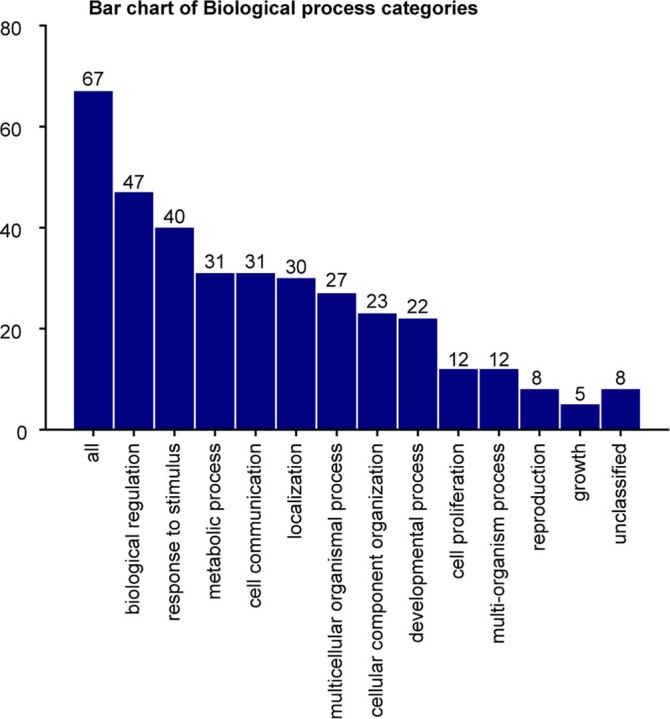
Annotation of genes shared by *KMT2A-*rearranged myeloid and lymphoid leukemias. Barplot showing biological categories of genes shared between the *KMT2A*-rearranged B-ALL and *KMT2A*-rearranged AML core cancer transcriptomes.

## Data Availability

Single-cell RNA sequences have been deposited in the European Nucleotide Archive (accession number ERP125305) and the European Genome-phenome Archive (accession number EGAD00001007854) (Figs. 2 and 4). DNA sequences of the lineage-switch case (PD38257a, PD38257b, PD38257c) have been deposited in the European Genome-phenome Archive under study ID EGAD00001007853 and RNA sequences in the NCBI Sequence Read Archive under project IDs PRJNA547947 and PRJNA547815 (Fig. 3). We used scRNA-seq data from developing bone marrow^9^, which are accessible through EMBL-European Bioinformatics Institute ArrayExpress and European Nucleotide Archive with accession codes E-MTAB-9389 and ERP125305. Scanpy h5ad objects with transformed counts are also available at https://fbm.cellatlas.io/. Bulk RNA-sequencing data on ELPs^18^ is available at GEO (GSE122982). TARGET leukemia RNA-sequencing data are available at dbGaP (phs000463, phs000464 and phs000465). St. Jude’s leukemia RNA-sequencing data were accessed via the St. Jude Cloud (https://stjudecloud.github.io/docs/citing-stjude-cloud/).

## Code availability

Jupyter Notebook (v6.4.0) for processing single-cell data, including Cell Ranger-filtered count data and steps to reproduce Figs. 1 and 2, is available at https://github.com/kheleon/leukemia-paper.
