# Supplementary material for: Single-cell transcriptomics reveals a distinct developmental state of KMT2A-rearranged infant B-cell acute lymphoblastic leukemia
Source: Nat Med. Author manuscript; Available in PMC 2022 Apr 26. (PMC9018413; doi:10.1038/s41591-022-01720-7)
Supplement: Reporting Summary [file EMS144370-supplement-Reporting_Summary.pdf]

nature research

Corresponding author(s): Simon Bomken, Jack Bartram, Muzlifah Haniffa, Sam Behjati

Last updated by author(s): 29/12/2021

Reporting Summary

Nature Research wishes to improve the reproducibility of the work that we publish. This form provides structure for consistency and transparency in reporting. For further information on Nature Research policies, see Authors & Referees and the Editorial Policy Checklist.

Statistics

For all statistical analyses, confirm that the following items are present in the figure legend, table legend, main text, or Methods section.

n/a	Confirmed	
☐	☒	The exact sample size (n) for each experimental group/condition, given as a discrete number and unit of measurement
☐	☒	A statement on whether measurements were taken from distinct samples or whether the same sample was measured repeatedly
☐	☒	The statistical test(s) used AND whether they are one- or two-sided<br>*Only common tests should be described solely by name; describe more complex techniques in the Methods section.*
☐	☒	A description of all covariates tested
☐	☒	A description of any assumptions or corrections, such as tests of normality and adjustment for multiple comparisons
☐	☒	A full description of the statistical parameters including central tendency (e.g. means) or other basic estimates (e.g. regression coefficient) AND variation (e.g. standard deviation) or associated estimates of uncertainty (e.g. confidence intervals)
☐	☒	For null hypothesis testing, the test statistic (e.g. F, t, r) with confidence intervals, effect sizes, degrees of freedom and P value noted<br>*Give P values as exact values whenever suitable.*
☐	☒	For Bayesian analysis, information on the choice of priors and Markov chain Monte Carlo settings
☒	☐	For hierarchical and complex designs, identification of the appropriate level for tests and full reporting of outcomes
☐	☒	Estimates of effect sizes (e.g. Cohen's d, Pearson's r), indicating how they were calculated

Our web collection on statistics for biologists contains articles on many of the points above.

Software and code

Policy information about availability of computer code

Data collection	Code availability statement: Jupyter notebook for processing single cell data, including cellranger filtered counts data and steps to reproduce Figure 1 and 2, is available at https://github.com/kheleon/leukemia-paper.<br><br>Raw fastq files for scRNA-seq data for P1_iALL,P2_iALL, P9_iAML were processed with Cell Ranger v2.0.2 pipeline and the rest of the samples were processed at later time point with Cell Ranger v3.0.2 pipeline.<br>Ambient mRNA contamination was removed with SoupX package v 1.4.8 in R .<br>Demultiplexing of P1_iALL/P2_iALL, P3_iALL/P10_iAML and P5_iALL/InfALLclassSwitch was performed with souporcell package v2.0.<br>Gene expression matrices were further processed<br><br>Flow cytometry data were acquired on a BD FACSAria running DIVA v.8.
Data analysis	scRANseq Dimensional reduction and clustering were performed in python with scanpy package v1.4.4.post1.<br>Bulk RNAseq data were quantified and mapped with Salmon<br>Transcript-level estimates were summarised with tximport package v 1.14.2 in R<br>Deconvolution of bulk RNAseq data used tensorflow framework v1.14.0<br>Differential gene expression analysis was performed using DESeq2 package v1.26.0 in R<br>Gene ontology analysis was performed using WebGestalt (WEB-based Gene SeT AnaLysis Toolkit)<br>DNA sequences were aligned to the GRCh37d5 reference genome by the Burrows-Wheeler algorithm (BWA-MEM)<br>Variant calling used the following algorithms: CaVEMan, PINDEL for insertions/deletions, ASCAT, Battenberg and BRASS<br>Jbrowse was used to visualize all shared substitutions<br>Mutational signature analysis used the SigFit algorithm and the COSMIC reference database of mutational signatures<br><br>Flow cytometry data were analysed using FlowJo (v.10.6.2, BD Biosciences).

For manuscripts utilizing custom algorithms or software that are central to the research but not yet described in published literature, software must be made available to editors/reviewers. We strongly encourage code deposition in a community repository (e.g. GitHub). See the Nature Research guidelines for submitting code & software for further information.

Data

Policy information about availability of data

All manuscripts must include a data availability statement. This statement should provide the following information, where applicable:

- Accession codes, unique identifiers, or web links for publicly available datasets
- A list of figures that have associated raw data
- A description of any restrictions on data availability

Data availability statement
Single cell RNA sequences have been deposited in the European Nucleotide Archive (accession number ERP125305) and in the European Genome-phenome Archive (accession number EGAD00001007854) (Figure 2, Figure 4). DNA sequences of the lineage switch case (PD38257a to c) have been deposited in the European Genome-phenome Archive under study ID EGAD00001007853 and RNA sequences in the NCBI Sequence Read Archive under project IDs PRJNA547947 and PRJNA547815 (Figure 3).
We used single cell RNA sequencing data from developing bone marrow (Jardine et al., 2021); accessible through EMBL-EBI ArrayExpress and ENA with accession codes E-MTAB-9389 and ERP125305. Scanpy h5ad objects with transformed counts are also available at https://fbm.cellatlas.io/. Bulk RNA sequencing data on ELPs (O'Byrne et al. 2019) is available at GEO (GSE122982). TARGET leukemia RNAsequencing data are available at dbGaP (phs000463, phs000464 and phs000465). St Jude's leukemia RNAsequencing data were accessed via the St Jude cloud (https://stjudecloud.github.io/docs/citing-stjude-cloud/).

Field-specific reporting

Please select the one below that is the best fit for your research. If you are not sure, read the appropriate sections before making your selection.

☒ Life sciences ☐ Behavioural & social sciences ☐ Ecological, evolutionary & environmental sciences

For a reference copy of the document with all sections, see nature.com/documents/nr-reporting-summary-flat.pdf

Life sciences study design

All studies must disclose on these points even when the disclosure is negative.

Sample size	Sample size calculations were not performed.<br>For bulk RNAseq data, n=1,665 bulk transcriptomes across two large cohorts was considered representative of the spectrum of childhood ALL and AML.<br>For scRNAseq data, as per the Human Cell Atlas white paper (https://www.humancellatlas.org/wp-content/uploads/2019/11/HCA_WhitePaper_18Oct2017-copyright.pdf), sample size for scRNAseq was determined by recent experience using these technologies in relevant tissues. In Jardine et al 2021, reproducible data were provided from n=4 trisomy 21 BM samples. We were able to access 6 KMT2A-rearranged infant ALL samples, and so generated data from n=6.<br>Flow cytometry was used to validate expression signatures . We reasoned that as this is a well-defined genetic subgroup of leukamia with a consistent transcriptional signature, n=3-6 would suffice.
Data exclusions	Deconvolution results for data points with only one case per disease subtype were excluded from the analysis (Figure 1). This criterion was pre-established as our goal was to survey the entire spectrum of childhood leukemia, rather than draw attention to signals in unique cases that could not be readily validated.
Replication	Reproducibility of key experimental findings was confirmed by using orthogonal approaches.<br>For the ELP signal in KMT2A rearranged leukemia, we used two independent data cohorts (Target and St Jude's) for cell signal analysis, n=52<br>We also generated our own scRNAseq data, n=6<br>For the findings relating to co-expression of specific proteins, we used transcriptome data and generated flow cytometry data.<br>For the differential expression analyses, we used ELP signals from two independent data sets.
Randomization	No interventions were performed in this study, therefore randomization was not required. All cases with n=2 of more were used from the St Judes's and Target cohorts, and cases for scRNAseq were selected based on availability of stored material.
Blinding	Blinding was not required as there were no measurements or interpretation that could have been influenced by prior knowledge of results.

Reporting for specific materials, systems and methods

We require information from authors about some types of materials, experimental systems and methods used in many studies. Here, indicate whether each material, system or method listed is relevant to your study. If you are not sure if a list item applies to your research, read the appropriate section before selecting a response.

Materials & experimental systems

n/a	Involved in the study
☐	☒ Antibodies
☒	☐ Eukaryotic cell lines
☒	☐ Palaeontology
☐	☒ Animals and other organisms
☐	☒ Human research participants
☒	☐ Clinical data

Methods

n/a	Involved in the study
☒	☐ ChIP-seq
☐	☒ Flow cytometry
☒	☐ MRI-based neuroimaging

Antibodies

Antibodies used

Antibodies for immunophenotyping (Fig. S4) were (clone; supplier; catalog number; dilution) NG2 PE (9.2.27; BD Biosciences; 562415; 1:25)
FLT3 PEDazzle (BV10A4H2; Biolegend; 313319; 1:25)
CD10 PECy7 (HI10a; Biolegend; 312213; 1:50)
CD2 FITC (S5.2, BD Biosciences; 347404; 1:25)
CD3 FITC (SK7; BD Biosciences; 345763; 1:25)
CD14 FITC (MφP9; BD Biosciences; 345784; 1:25)
CD16 FITC (NKP15; BD Biosciences; 335035; 1:25)
CD56 FITC (NCAM16.2; BD Biosciences; 345811; 1:25)
CD235a FITC (GA-R2; BD Biosciences; 559943; 1:25)
CD38 PERCPCy5.5 (HB-7: Biolegend; 356614; 1:25)
CD45RA BV510 (HI100; BD Biosciences; 563031; 1:25)
CD7 BV650 M-T701; BD Biosciences; 740565; 1:50)
CD127 BUV737 (HIL-7R-M21; BD Biosciences; 612795; 1:25)
CD90 APC (5E10; Biolegend; 328114; 1:25)
CD19 AF700 (HIB19; Biolegend; 302226; 1:25)
CD34 APCCy7 (581; Biolegend; 343514; 1:25)
SEMA4A PE (T9-10; BD Biosciences; 564812; 1:25)
LILRB1 PE (GHI/75; Biolegend; 551053; 1:25)
CD19 FITC (4G7; BD Biosciences; 345776; 1:25)
ICOSLG BV510 (2D3/B7-H2l; BD Biosciences; 743006; 1:25)
CD72 APC (SF3; Biolegend; 316209; 1:25)

Validation

Antibodies were validated by the manufacturer. Our flow cytometry data adhere to the information standards for MIFlowCyt for Flow/Mass cytometry (https://onlinelibrary.wiley.com/doi/pdf/10.1002/cyto.a.20623).

Animals and other organisms

Policy information about studies involving animals; ARRIVE guidelines recommended for reporting animal research

Laboratory animals

Patient-derived xenografts (PDX) were generated by intrafemoral transplant of patient blood/BM into NOD.Cg-Prkdcscid Il2rgtm1Wjl/SzJ (NSG, Charles River Labs and bred in-house) mice (both sexes), age 8-10 weeks old were transplanted intra-femorally under isoflurane anaesthesia with 10 million cells. Mice were killed when any predetermined humane endpoint was reached.

Wild animals

The study did not involve wild animals.

Field-collected samples

The study did not involve field-collected samples.

Ethics oversight

Patient-derived xenograft samples (PDX) were generated in accordance with the UK Animals (Scientific Procedures) Act 1986 under project licences PPL60/4552 and PPL60/4222 following institutional ethical review (Newcastle & North Tyneside Research Ethics Committee).

Note that full information on the approval of the study protocol must also be provided in the manuscript.

Human research participants

Policy information about studies involving human research participants

Population characteristics

Original data from 14 children with leukemia are presented: age median 6 months, range 0-55 months; gender 6 male and 8 female; disease KMT2A-rearranged infant ALL in 11 cases, ETV6-RUNX1 fusion ALL in 1 case, infant AML with t(1;22) and t(6;11) in 2 cases, and lineage-switched KMT2A-rearranged AML in 1 case who had prior KMT2A-rearranged infant ALL. Diagnostic samples were obtained from 13 children, day 8 from 2 children and relapse samples from 2 children. Children were treated according to contemporary UK practice.

Recruitment

Samples were obtained from Newcastle Biobank and GOSH diagnostic archives. All cases of infant leukemia were approached. A

Recruitment	limited number of families with more common diagnoses e.g. ETV6-RUNX1 ALL were approached. No biological connection between willingness to participate and leukemia transcriptome or surface protein expression is likely.
Ethics oversight	Patient blood/bone marrow samples were obtained from the Newcastle Biobank (as approved by Newcastle & North Tyneside 1 Research Ethics Committee, reference 17/NE/0361) or GOSH diagnostic archives (as approved by National Research Ethics Service Committee London Brent, reference 16/LO/0960). Informed consent was obtained from all participants.

Note that full information on the approval of the study protocol must also be provided in the manuscript.

Flow Cytometry

Plots

Confirm that:

☒ The axis labels state the marker and fluorochrome used (e.g. CD4-FITC).

☒ The axis scales are clearly visible. Include numbers along axes only for bottom left plot of group (a 'group' is an analysis of identical markers).

☒ All plots are contour plots with outliers or pseudocolor plots.

☒ A numerical value for number of cells or percentage (with statistics) is provided.

Methodology

Sample preparation	Adherent material was removed from fetal femur and bone was cut into small pieces before grinding with a pestle and mortar. Flow buffer (PBS containing 5% (v/v) FBS and 2 mM EDTA) was added to reduce clumping. The suspension was filtered with a 70µm filter then centrifuged for 5 min at 500g. The supernatant was removed before cells were treated with 1x RBC lysis buffer (eBioscience) for 5 min at room temperature and washed once with Flow Buffer before counting.
Instrument	Flow sorting was performed on a BD FACSAriaTM Fusion instrument
Software	FlowJoV10.4.1
Cell population abundance	Abundance of CD45 positive and negative fractions for droplet single sequencing were determined by cell counting post sort.
Gating strategy	As mentioned in Methods and shown in Extended Data Figure 1e/6c, for all flow experiments, cells were gates based on FSC/SSC, live (DAPI negative set based on unstained cells from the sample sample) and single cells (FSC-H/FSC-A). For single cell sequencing, the 'positive' gate was set between the middle of positive and negative staining to the edge of plot, and 'negative' was set to everything else to ensure that all cells were accounted for. For validation experiments (Smart-Seq2, cytospins and culture sorts), gates were set over the bulk of the positive staining excluding the edges of staining. Our flow cytometry data adhere to the information standards for Flow cytometry (https://onlinelibrary.wiley.com/doi/pdf/10.1002/cyto.a.20623).

☒ Tick this box to confirm that a figure exemplifying the gating strategy is provided in the Supplementary Information.
