# Supplementary material for: Single-cell transcriptomics reveals a distinct developmental state of KMT2A-rearranged infant B-cell acute lymphoblastic leukemia
Source: Nat Med. Author manuscript; Available in PMC 2022 Apr 26. (PMC9018413; doi:10.1038/s41591-022-01720-7)
Supplement: Supplementary Figure 1 and legends for supplementary tables. [file EMS144370-supplement-Supplementary_Figure_1_and_legends_for_supplementary_tables_.pdf]

nature portfolio

https://doi.org/10.1038/s41591-022-01720-7

## Supplementary information

# Single-cell transcriptomics reveals a distinct developmental state of *KMT2A*-rearranged infant B-cell acute lymphoblastic leukemia

In the format provided by the authors and unedited

# Single-cell transcriptomics reveals a distinct developmental state of *KMT2A*-rearranged infant B-cell acute lymphoblastic leukemia

Eleonora Khabirova[1]†, Laura Jardine[2,3]†, Tim H. H. Coorens[1], Simone Webb[2], Taryn D. Treger[1,4,5], Justin Engelbert[2], Tarryn Porter[1], Elena Prigmore[1], Grace Collord[1,6,7], Alice Piapi[8,12], Sarah A. Teichmann[1], Sarah Inglott[12], Owen Williams[8], Olaf Heidenreich[9,10], Matthew D. Young[1], Karin Straathof[8,12], Simon Bomken[10,11*], Jack Bartram[8,12*], Muzlifah Haniffa[1,2,13*], Sam Behjati[1,4,5*]

Correspondence to: sb31@sanger.ac.uk, m.a.haniffa@newcastle.ac.uk, Jack.Bartram@gosh.nhs.uk, s.n.bomken@newcastle.ac.uk

## Supplementary Information

**Supplementary Fig. 1.** Cell signal analysis of 1,665 leukemia transcriptomes (per case). Heatmaps showing cell signals of fetal bone marrow cells (y-axis) in human leukaemia bulk transcriptomes for B-ALL genetic subtypes and additional cancers in St Jude's and TARGET cohorts. Each row represents a single case. For signals averaged across genetic subgroups see **Fig. 1B**.

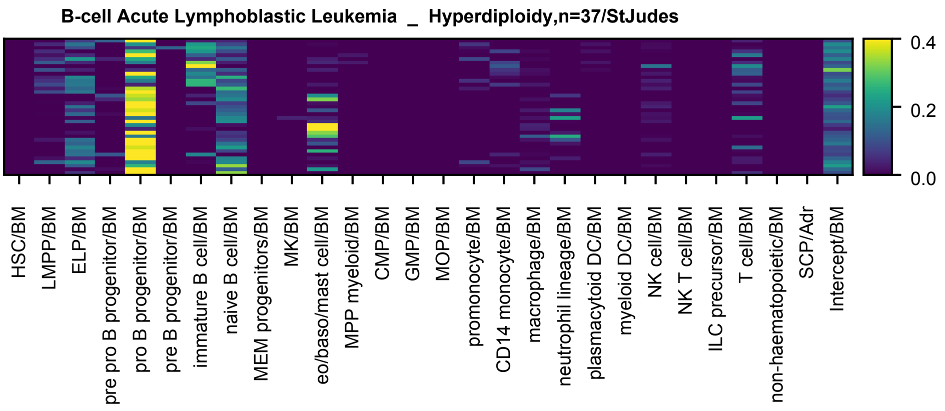
B-cell Acute Lymphoblastic Leukemia _ Hyperdiploidy,n=37/StJudes
HSC/BM
LMPP/BM
ELP/BM
pre pro B progenitor/BM
pro B progenitor/BM
pre B progenitor/BM
immature B cell/BM
naive B cell/BM
MEM progenitors/BM
MK/BM
eo/baso/mast cell/BM
MPP myeloid/BM
CMP/BM
GMP/BM
MOP/BM
promonocyte/BM
CD14 monocyte/BM
macrophage/BM
neutrophil lineage/BM
plasmacytoid DC/BM
myeloid DC/BM
NK cell/BM
NK T cell/BM
ILC precursor/BM
T cell/BM
non-haematopoietic/BM
SCP/Adr
Intercept/BM
0.4
0.2
0.0

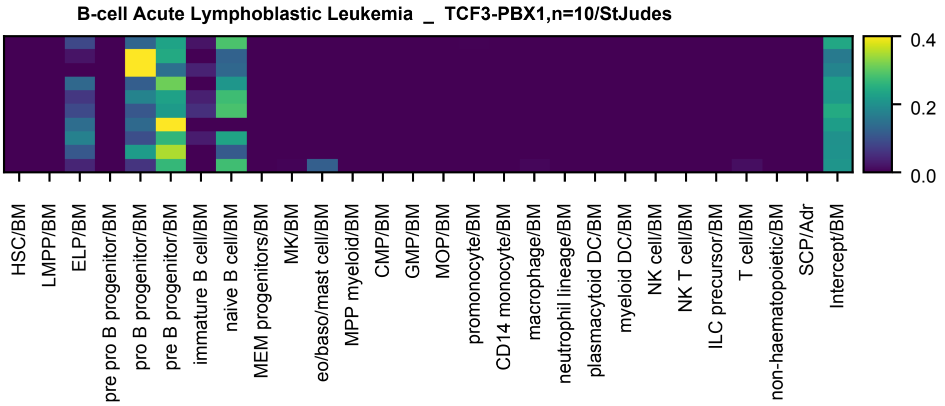
B-cell Acute Lymphoblastic Leukemia _ TCF3-PBX1,n=10/StJudes
HSC/BM
LMPP/BM
ELP/BM
pre pro B progenitor/BM
pro B progenitor/BM
pre B progenitor/BM
immature B cell/BM
naive B cell/BM
MEM progenitors/BM
MK/BM
eo/baso/mast cell/BM
MPP myeloid/BM
CMP/BM
GMP/BM
MOP/BM
promonocyte/BM
CD14 monocyte/BM
macrophage/BM
neutrophil lineage/BM
plasmacytoid DC/BM
myeloid DC/BM
NK cell/BM
NK T cell/BM
ILC precursor/BM
T cell/BM
non-haematopoietic/BM
SCP/Adr
Intercept/BM
0.4
0.2
0.0

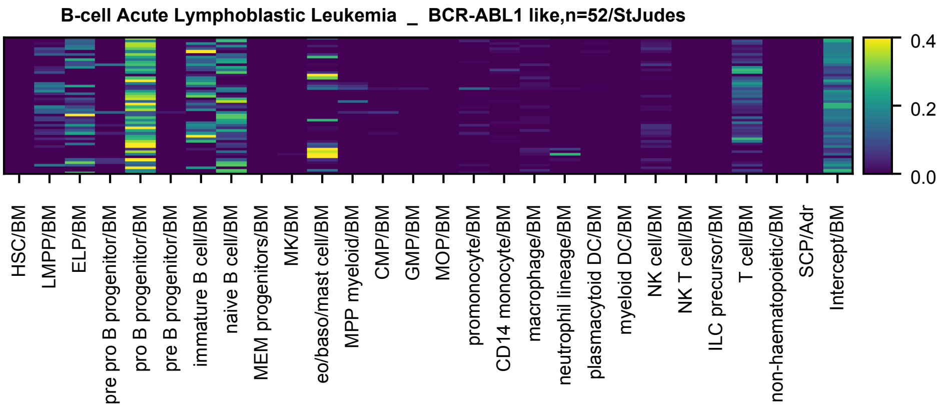
B-cell Acute Lymphoblastic Leukemia _ BCR-ABL1 like,n=52/StJudes
HSC/BM
LMPP/BM
ELP/BM
pre pro B progenitor/BM
pro B progenitor/BM
pre B progenitor/BM
immature B cell/BM
naive B cell/BM
MEM progenitors/BM
MK/BM
eo/baso/mast cell/BM
MPP myeloid/BM
CMP/BM
GMP/BM
MOP/BM
promonocyte/BM
CD14 monocyte/BM
macrophage/BM
neutrophil lineage/BM
plasmacytoid DC/BM
myeloid DC/BM
NK cell/BM
NK T cell/BM
ILC precursor/BM
T cell/BM
non-haematopoietic/BM
SCP/Adr
Intercept/BM
0.4
0.2
0.0

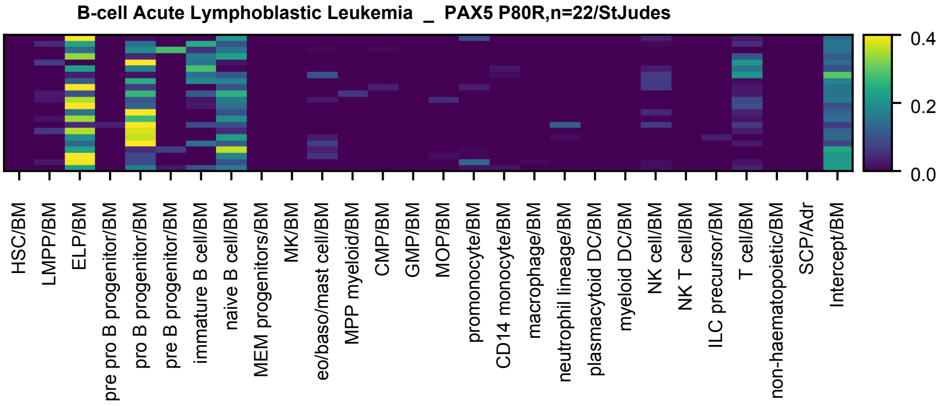
B-cell Acute Lymphoblastic Leukemia _ PAX5 P80R,n=22/StJudes
HSC/BM
LMPP/BM
ELP/BM
pre pro B progenitor/BM
pro B progenitor/BM
pre B progenitor/BM
immature B cell/BM
naive B cell/BM
MEM progenitors/BM
MK/BM
eo/baso/mast cell/BM
MPP myeloid/BM
CMP/BM
GMP/BM
MOP/BM
promonocyte/BM
CD14 monocyte/BM
macrophage/BM
neutrophil lineage/BM
plasmacytoid DC/BM
myeloid DC/BM
NK cell/BM
NK T cell/BM
ILC precursor/BM
T cell/BM
non-haematopoietic/BM
SCP/Adr
Intercept/BM
0.4
0.2
0.0

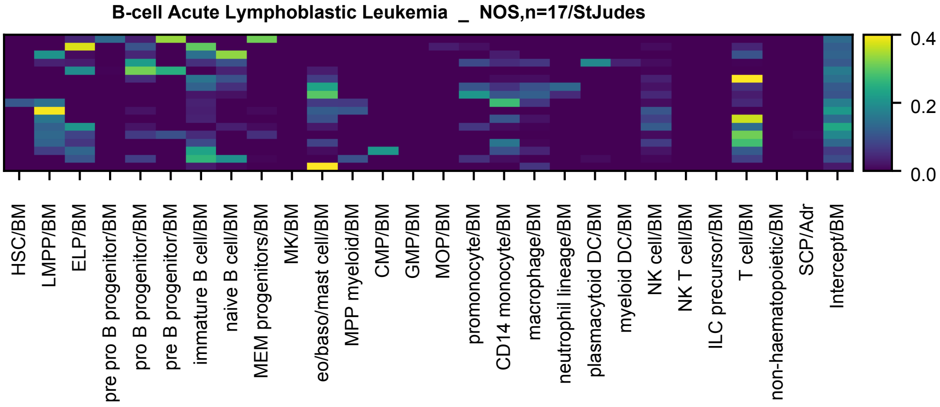
B-cell Acute Lymphoblastic Leukemia _ NOS,n=17/StJudes
0.4
0.2
0.0
HSC/BM
LMPP/BM
ELP/BM
pre pro B progenitor/BM
pro B progenitor/BM
pre B progenitor/BM
immature B cell/BM
naive B cell/BM
MEM progenitors/BM
MK/BM
eo/baso/mast cell/BM
MPP myeloid/BM
CMP/BM
GMP/BM
MOP/BM
promonocyte/BM
CD14 monocyte/BM
macrophage/BM
neutrophil lineage/BM
plasmacytoid DC/BM
myeloid DC/BM
NK cell/BM
NK T cell/BM
ILC precursor/BM
T cell/BM
non-haematopoietic/BM
SCP/Adr
Intercept/BM

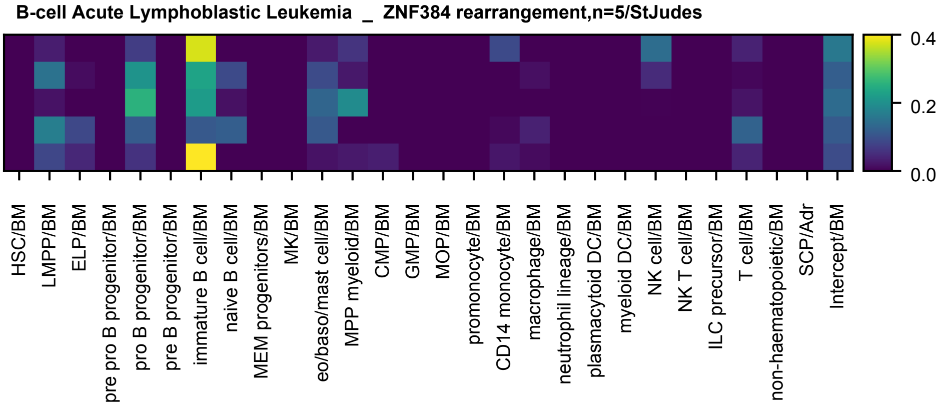
B-cell Acute Lymphoblastic Leukemia _ ZNF384 rearrangement,n=5/StJudes
0.4
0.2
0.0
HSC/BM
LMPP/BM
ELP/BM
pre pro B progenitor/BM
pro B progenitor/BM
pre B progenitor/BM
immature B cell/BM
naive B cell/BM
MEM progenitors/BM
MK/BM
eo/baso/mast cell/BM
MPP myeloid/BM
CMP/BM
GMP/BM
MOP/BM
promonocyte/BM
CD14 monocyte/BM
macrophage/BM
neutrophil lineage/BM
plasmacytoid DC/BM
myeloid DC/BM
NK cell/BM
NK T cell/BM
ILC precursor/BM
T cell/BM
non-haematopoietic/BM
SCP/Adr
Intercept/BM

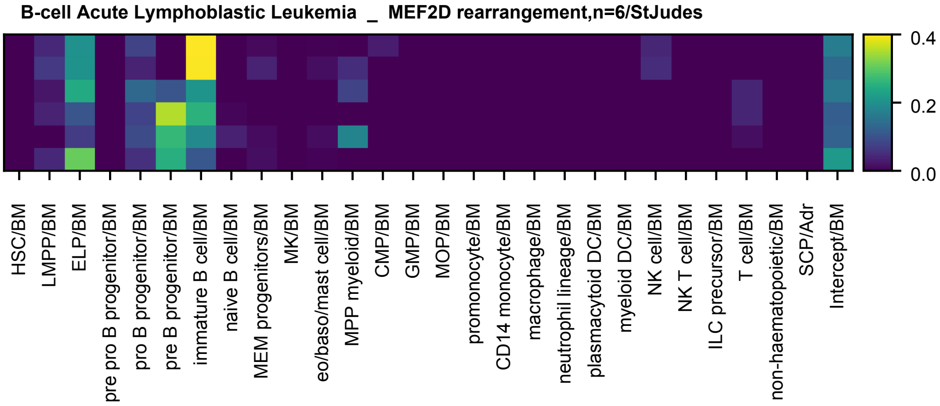
B-cell Acute Lymphoblastic Leukemia _ MEF2D rearrangement,n=6/StJudes
0.4
0.2
0.0
HSC/BM
LMPP/BM
ELP/BM
pre pro B progenitor/BM
pro B progenitor/BM
pre B progenitor/BM
immature B cell/BM
naive B cell/BM
MEM progenitors/BM
MK/BM
eo/baso/mast cell/BM
MPP myeloid/BM
CMP/BM
GMP/BM
MOP/BM
promonocyte/BM
CD14 monocyte/BM
macrophage/BM
neutrophil lineage/BM
plasmacytoid DC/BM
myeloid DC/BM
NK cell/BM
NK T cell/BM
ILC precursor/BM
T cell/BM
non-haematopoietic/BM
SCP/Adr
Intercept/BM

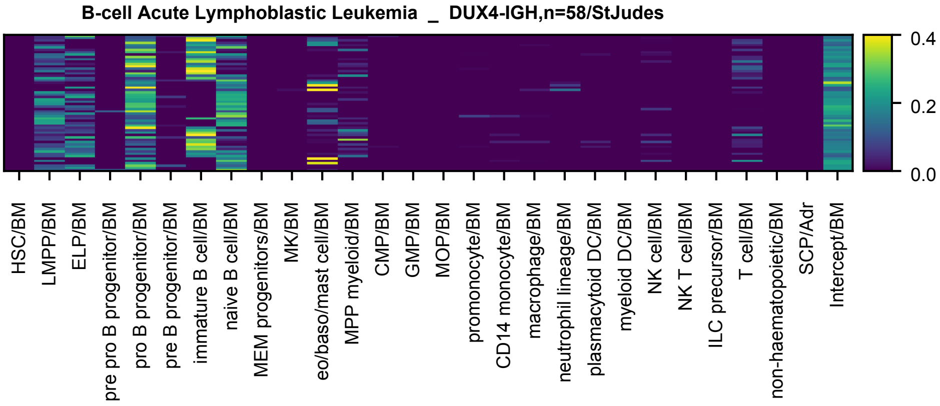
B-cell Acute Lymphoblastic Leukemia _ DUX4-IGH,n=58/StJudes
0.4
0.2
0.0
HSC/BM
LMPP/BM
ELP/BM
pre pro B progenitor/BM
pro B progenitor/BM
pre B progenitor/BM
immature B cell/BM
naive B cell/BM
MEM progenitors/BM
MK/BM
eo/baso/mast cell/BM
MPP myeloid/BM
CMP/BM
GMP/BM
MOP/BM
promonocyte/BM
CD14 monocyte/BM
macrophage/BM
neutrophil lineage/BM
plasmacytoid DC/BM
myeloid DC/BM
NK cell/BM
NK T cell/BM
ILC precursor/BM
T cell/BM
non-haematopoietic/BM
SCP/Adr
Intercept/BM

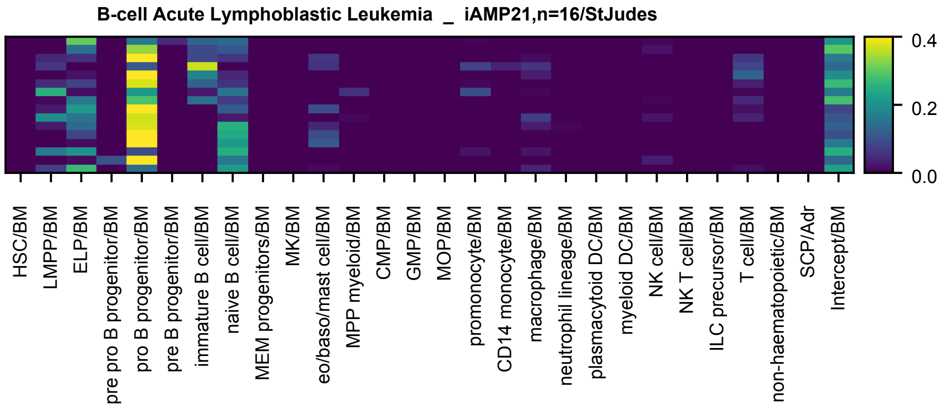
B-cell Acute Lymphoblastic Leukemia _ iAMP21,n=16/StJudes
0.4
0.2
0.0
HSC/BM
LMPP/BM
ELP/BM
pre pro B progenitor/BM
pro B progenitor/BM
pre B progenitor/BM
immature B cell/BM
naive B cell/BM
MEM progenitors/BM
MK/BM
eo/baso/mast cell/BM
MPP myeloid/BM
CMP/BM
GMP/BM
MOP/BM
promonocyte/BM
CD14 monocyte/BM
macrophage/BM
neutrophil lineage/BM
plasmacytoid DC/BM
myeloid DC/BM
NK cell/BM
NK T cell/BM
ILC precursor/BM
T cell/BM
non-haematopoietic/BM
SCP/Adr
Intercept/BM

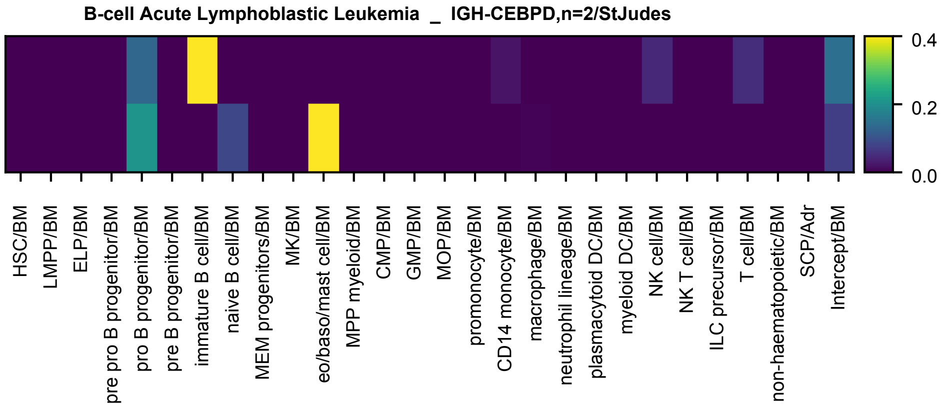
B-cell Acute Lymphoblastic Leukemia _ IGH-CEBPD,n=2/StJudes
0.4
0.2
0.0
HSC/BM
LMPP/BM
ELP/BM
pre pro B progenitor/BM
pro B progenitor/BM
pre B progenitor/BM
immature B cell/BM
naive B cell/BM
MEM progenitors/BM
MK/BM
eo/baso/mast cell/BM
MPP myeloid/BM
CMP/BM
GMP/BM
MOP/BM
promonocyte/BM
CD14 monocyte/BM
macrophage/BM
neutrophil lineage/BM
plasmacytoid DC/BM
myeloid DC/BM
NK cell/BM
NK T cell/BM
ILC precursor/BM
T cell/BM
non-haematopoietic/BM
SCP/Adr
Intercept/BM

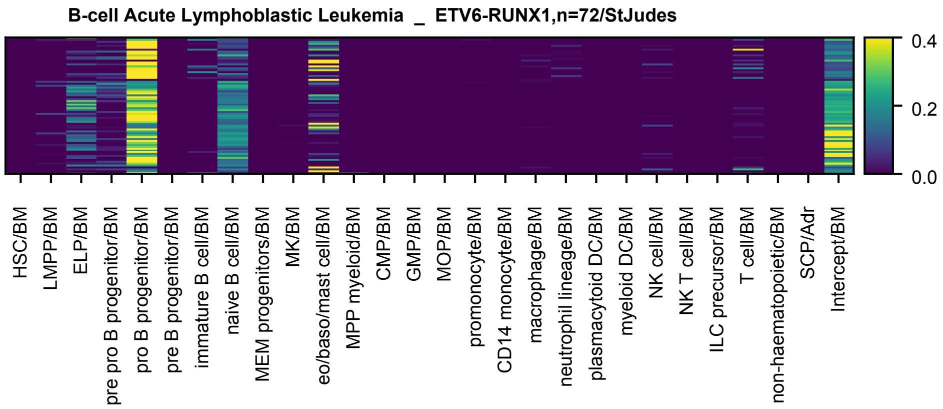
B-cell Acute Lymphoblastic Leukemia _ ETV6-RUNX1,n=72/StJudes
0.4
0.2
0.0
HSC/BM
LMPP/BM
ELP/BM
pre pro B progenitor/BM
pro B progenitor/BM
pre B progenitor/BM
immature B cell/BM
naive B cell/BM
MEM progenitors/BM
MK/BM
eo/baso/mast cell/BM
MPP myeloid/BM
CMP/BM
GMP/BM
MOP/BM
promonocyte/BM
CD14 monocyte/BM
macrophage/BM
neutrophil lineage/BM
plasmacytoid DC/BM
myeloid DC/BM
NK cell/BM
NK T cell/BM
ILC precursor/BM
T cell/BM
non-haematopoietic/BM
SCP/Adr
Intercept/BM

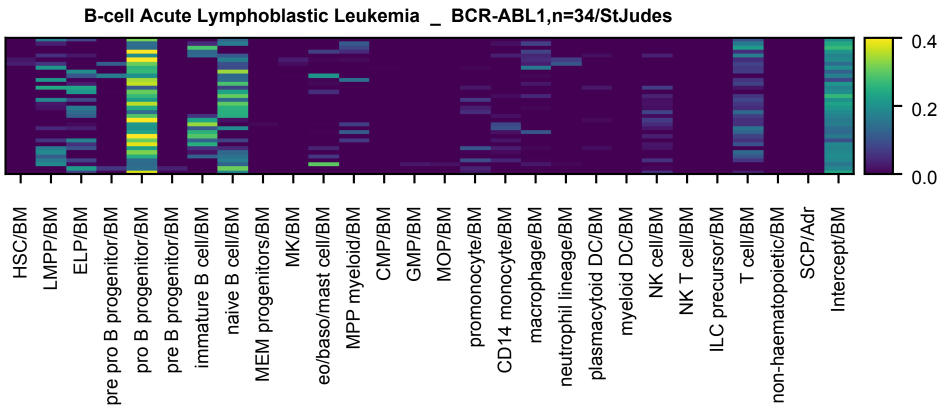
B-cell Acute Lymphoblastic Leukemia _ BCR-ABL1,n=34/StJudes
0.4
0.2
0.0
HSC/BM
LMPP/BM
ELP/BM
pre pro B progenitor/BM
pro B progenitor/BM
pre B progenitor/BM
immature B cell/BM
naive B cell/BM
MEM progenitors/BM
MK/BM
eo/baso/mast cell/BM
MPP myeloid/BM
CMP/BM
GMP/BM
MOP/BM
promonocyte/BM
CD14 monocyte/BM
macrophage/BM
neutrophil lineage/BM
plasmacytoid DC/BM
myeloid DC/BM
NK cell/BM
NK T cell/BM
ILC precursor/BM
T cell/BM
non-haematopoietic/BM
SCP/Adr
Intercept/BM

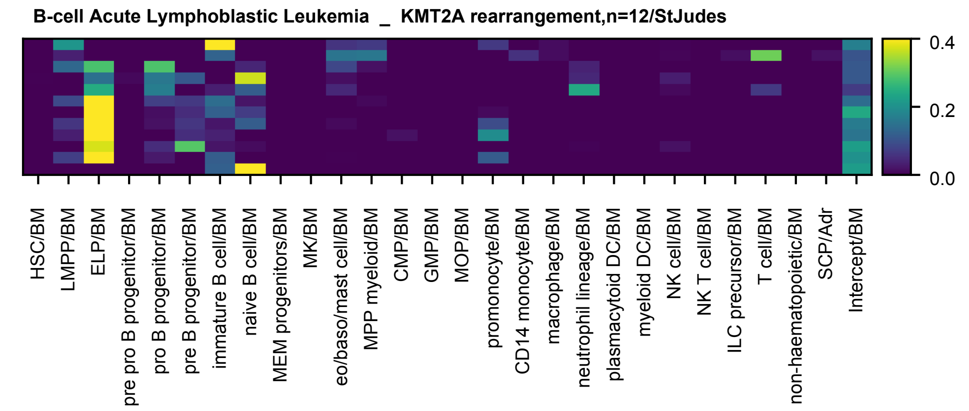
B-cell Acute Lymphoblastic Leukemia _ KMT2A rearrangement,n=12/StJudes
0.4
0.2
0.0
HSC/BM
LMPP/BM
ELP/BM
pre pro B progenitor/BM
pro B progenitor/BM
pre B progenitor/BM
immature B cell/BM
naive B cell/BM
MEM progenitors/BM
MK/BM
eo/baso/mast cell/BM
MPP myeloid/BM
CMP/BM
GMP/BM
MOP/BM
promonocyte/BM
CD14 monocyte/BM
macrophage/BM
neutrophil lineage/BM
plasmacytoid DC/BM
myeloid DC/BM
NK cell/BM
NK T cell/BM
ILC precursor/BM
T cell/BM
non-haematopoietic/BM
SCP/Adr
Intercept/BM

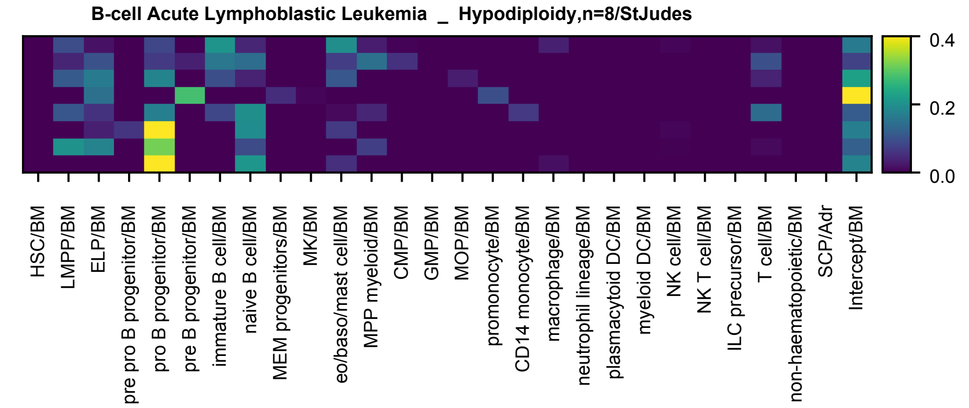
B-cell Acute Lymphoblastic Leukemia _ Hypodiploidy,n=8/StJudes
0.4
0.2
0.0
HSC/BM
LMPP/BM
ELP/BM
pre pro B progenitor/BM
pro B progenitor/BM
pre B progenitor/BM
immature B cell/BM
naive B cell/BM
MEM progenitors/BM
MK/BM
eo/baso/mast cell/BM
MPP myeloid/BM
CMP/BM
GMP/BM
MOP/BM
promonocyte/BM
CD14 monocyte/BM
macrophage/BM
neutrophil lineage/BM
plasmacytoid DC/BM
myeloid DC/BM
NK cell/BM
NK T cell/BM
ILC precursor/BM
T cell/BM
non-haematopoietic/BM
SCP/Adr
Intercept/BM

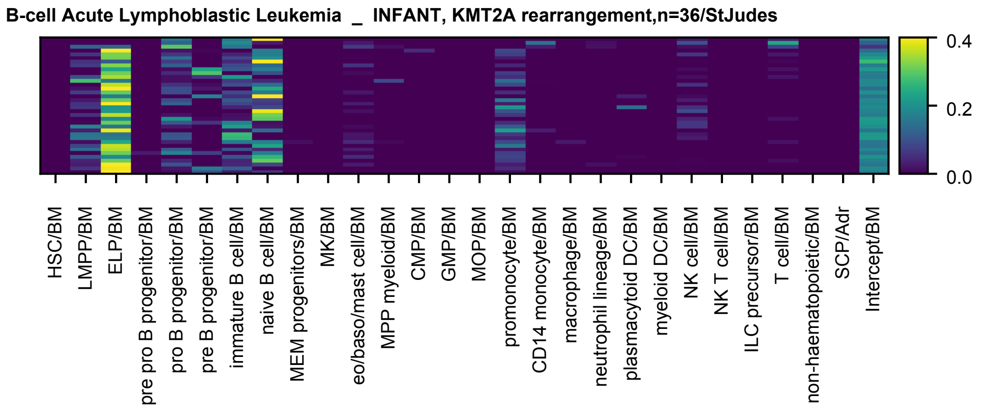
B-cell Acute Lymphoblastic Leukemia _ INFANT, KMT2A rearrangement,n=36/StJudes
0.4
0.2
0.0
HSC/BM
LMPP/BM
ELP/BM
pre pro B progenitor/BM
pro B progenitor/BM
pre B progenitor/BM
immature B cell/BM
naive B cell/BM
MEM progenitors/BM
MK/BM
eo/baso/mast cell/BM
MPP myeloid/BM
CMP/BM
GMP/BM
MOP/BM
promonocyte/BM
CD14 monocyte/BM
macrophage/BM
neutrophil lineage/BM
plasmacytoid DC/BM
myeloid DC/BM
NK cell/BM
NK T cell/BM
ILC precursor/BM
T cell/BM
non-haematopoietic/BM
SCP/Adr
Intercept/BM

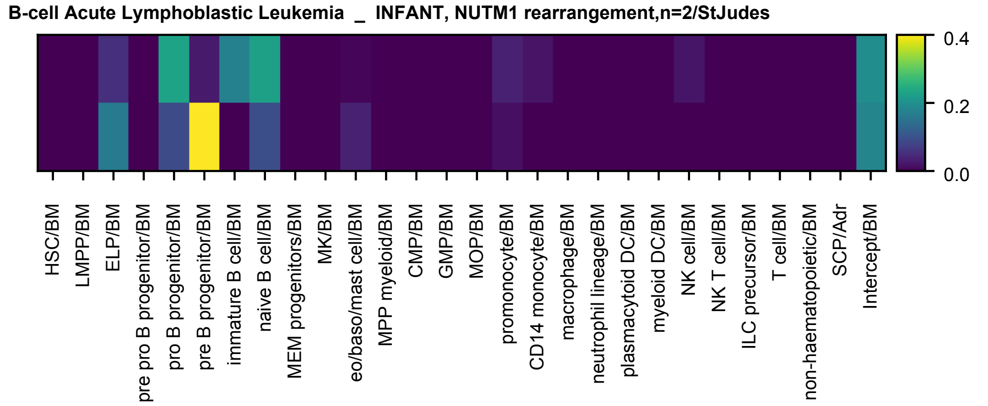
B-cell Acute Lymphoblastic Leukemia _ INFANT, NUTM1 rearrangement,n=2/StJudes
0.4
0.2
0.0
HSC/BM
LMPP/BM
ELP/BM
pre pro B progenitor/BM
pro B progenitor/BM
pre B progenitor/BM
immature B cell/BM
naive B cell/BM
MEM progenitors/BM
MK/BM
eo/baso/mast cell/BM
MPP myeloid/BM
CMP/BM
GMP/BM
MOP/BM
promonocyte/BM
CD14 monocyte/BM
macrophage/BM
neutrophil lineage/BM
plasmacytoid DC/BM
myeloid DC/BM
NK cell/BM
NK T cell/BM
ILC precursor/BM
T cell/BM
non-haematopoietic/BM
SCP/Adr
Intercept/BM

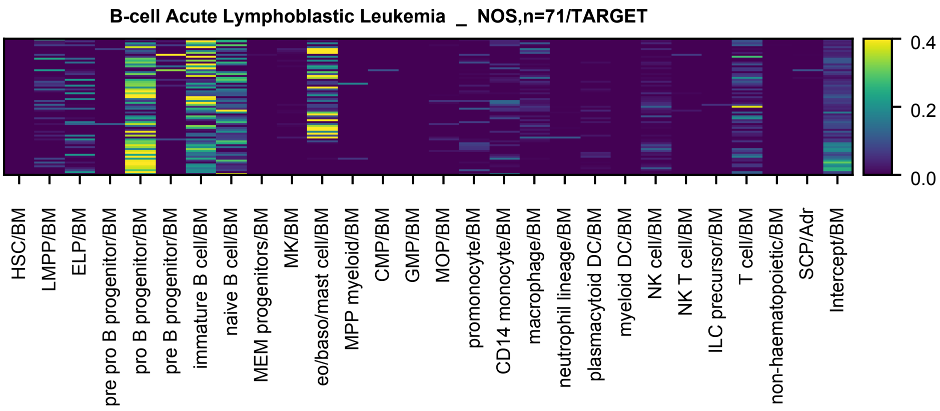
B-cell Acute Lymphoblastic Leukemia _ NOS,n=71/TARGET
0.4
0.2
0.0
HSC/BM
LMPP/BM
ELP/BM
pre pro B progenitor/BM
pro B progenitor/BM
pre B progenitor/BM
immature B cell/BM
naive B cell/BM
MEM progenitors/BM
MK/BM
eo/baso/mast cell/BM
MPP myeloid/BM
CMP/BM
GMP/BM
MOP/BM
promonocyte/BM
CD14 monocyte/BM
macrophage/BM
neutrophil lineage/BM
plasmacytoid DC/BM
myeloid DC/BM
NK cell/BM
NK T cell/BM
ILC precursor/BM
T cell/BM
non-haematopoietic/BM
SCP/Adr
Intercept/BM

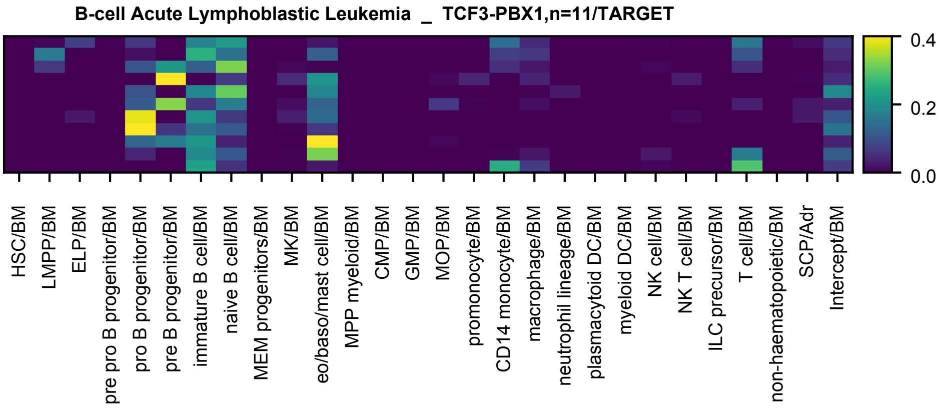
B-cell Acute Lymphoblastic Leukemia _ TCF3-PBX1,n=11/TARGET
0.4
0.2
0.0
HSC/BM
LMPP/BM
ELP/BM
pre pro B progenitor/BM
pro B progenitor/BM
pre B progenitor/BM
immature B cell/BM
naive B cell/BM
MEM progenitors/BM
MK/BM
eo/baso/mast cell/BM
MPP myeloid/BM
CMP/BM
GMP/BM
MOP/BM
promonocyte/BM
CD14 monocyte/BM
macrophage/BM
neutrophil lineage/BM
plasmacytoid DC/BM
myeloid DC/BM
NK cell/BM
NK T cell/BM
ILC precursor/BM
T cell/BM
non-haematopoietic/BM
SCP/Adr
Intercept/BM

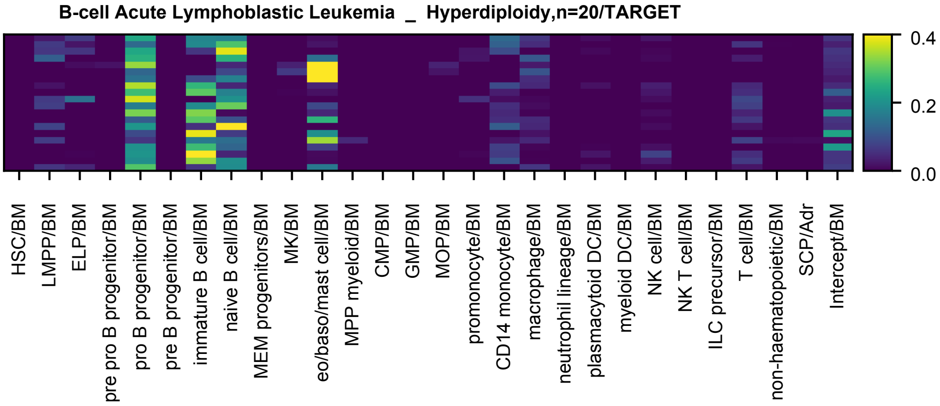
B-cell Acute Lymphoblastic Leukemia _ Hyperdiploidy,n=20/TARGET
0.4
0.2
0.0
HSC/BM
LMPP/BM
ELP/BM
pre pro B progenitor/BM
pro B progenitor/BM
pre B progenitor/BM
immature B cell/BM
naive B cell/BM
MEM progenitors/BM
MK/BM
eo/baso/mast cell/BM
MPP myeloid/BM
CMP/BM
GMP/BM
MOP/BM
promonocyte/BM
CD14 monocyte/BM
macrophage/BM
neutrophil lineage/BM
plasmacytoid DC/BM
myeloid DC/BM
NK cell/BM
NK T cell/BM
ILC precursor/BM
T cell/BM
non-haematopoietic/BM
SCP/Adr
Intercept/BM

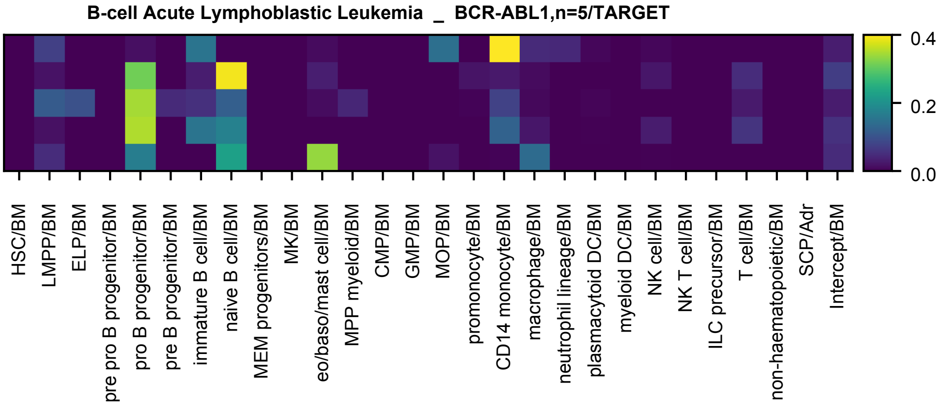
B-cell Acute Lymphoblastic Leukemia _ BCR-ABL1,n=5/TARGET
0.4
0.2
0.0
HSC/BM
LMPP/BM
ELP/BM
pre pro B progenitor/BM
pro B progenitor/BM
pre B progenitor/BM
immature B cell/BM
naive B cell/BM
MEM progenitors/BM
MK/BM
eo/baso/mast cell/BM
MPP myeloid/BM
CMP/BM
GMP/BM
MOP/BM
promonocyte/BM
CD14 monocyte/BM
macrophage/BM
neutrophil lineage/BM
plasmacytoid DC/BM
myeloid DC/BM
NK cell/BM
NK T cell/BM
ILC precursor/BM
T cell/BM
non-haematopoietic/BM
SCP/Adr
Intercept/BM

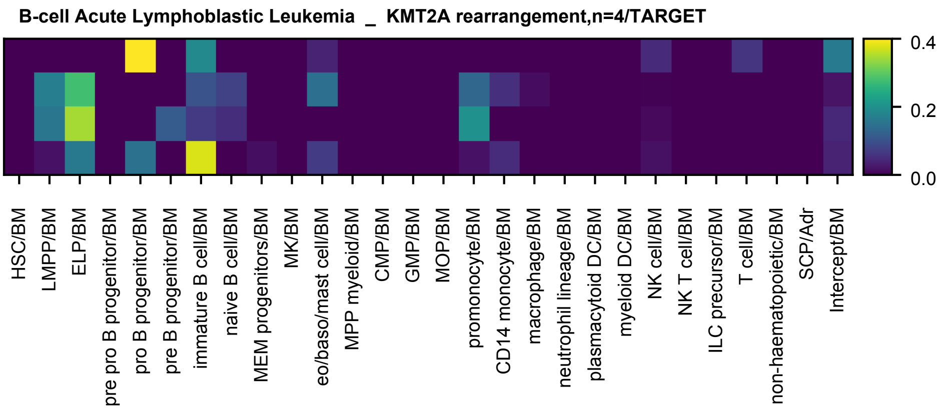
B-cell Acute Lymphoblastic Leukemia _ KMT2A rearrangement,n=4/TARGET
0.4
0.2
0.0
HSC/BM
LMPP/BM
ELP/BM
pre pro B progenitor/BM
pro B progenitor/BM
pre B progenitor/BM
immature B cell/BM
naive B cell/BM
MEM progenitors/BM
MK/BM
eo/baso/mast cell/BM
MPP myeloid/BM
CMP/BM
GMP/BM
MOP/BM
promonocyte/BM
CD14 monocyte/BM
macrophage/BM
neutrophil lineage/BM
plasmacytoid DC/BM
myeloid DC/BM
NK cell/BM
NK T cell/BM
ILC precursor/BM
T cell/BM
non-haematopoietic/BM
SCP/Adr
Intercept/BM

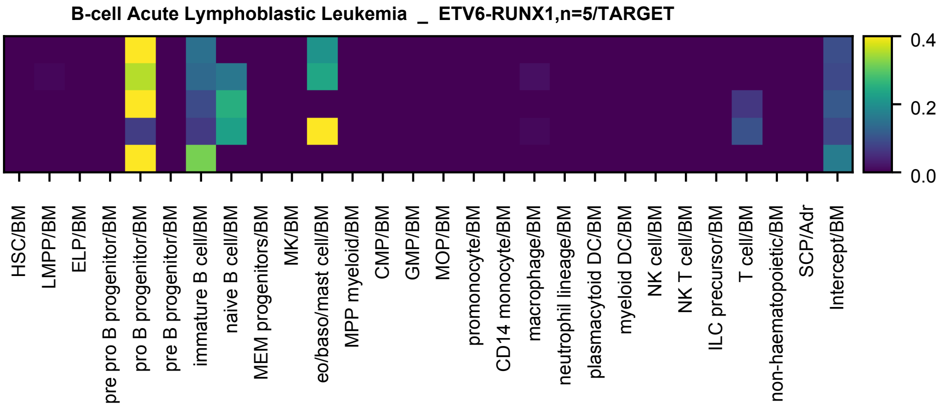
B-cell Acute Lymphoblastic Leukemia _ ETV6-RUNX1,n=5/TARGET
0.4
0.2
0.0
HSC/BM
LMPP/BM
ELP/BM
pre pro B progenitor/BM
pro B progenitor/BM
pre B progenitor/BM
immature B cell/BM
naive B cell/BM
MEM progenitors/BM
MK/BM
eo/baso/mast cell/BM
MPP myeloid/BM
CMP/BM
GMP/BM
MOP/BM
promonocyte/BM
CD14 monocyte/BM
macrophage/BM
neutrophil lineage/BM
plasmacytoid DC/BM
myeloid DC/BM
NK cell/BM
NK T cell/BM
ILC precursor/BM
T cell/BM
non-haematopoietic/BM
SCP/Adr
Intercept/BM

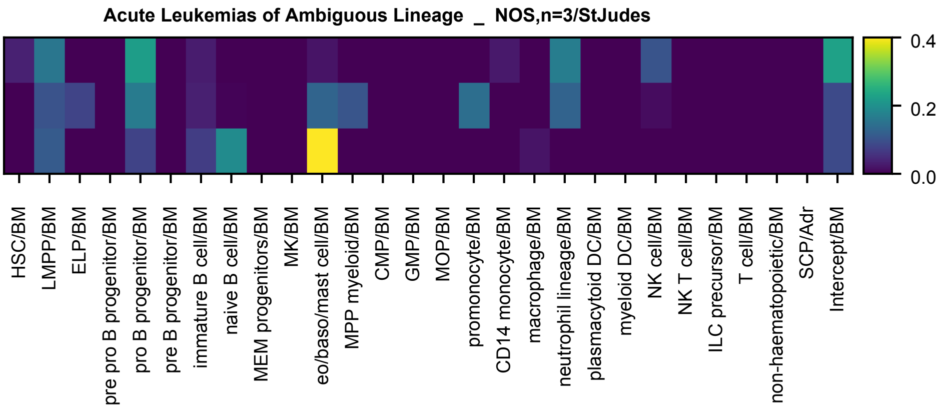
Acute Leukemias of Ambiguous Lineage _ NOS,n=3/StJudes
0.4
0.2
0.0
HSC/BM
LMPP/BM
ELP/BM
pre pro B progenitor/BM
pro B progenitor/BM
pre B progenitor/BM
immature B cell/BM
naive B cell/BM
MEM progenitors/BM
MK/BM
eo/baso/mast cell/BM
MPP myeloid/BM
CMP/BM
GMP/BM
MOP/BM
promonocyte/BM
CD14 monocyte/BM
macrophage/BM
neutrophil lineage/BM
plasmacytoid DC/BM
myeloid DC/BM
NK cell/BM
NK T cell/BM
ILC precursor/BM
T cell/BM
non-haematopoietic/BM
SCP/Adr
Intercept/BM

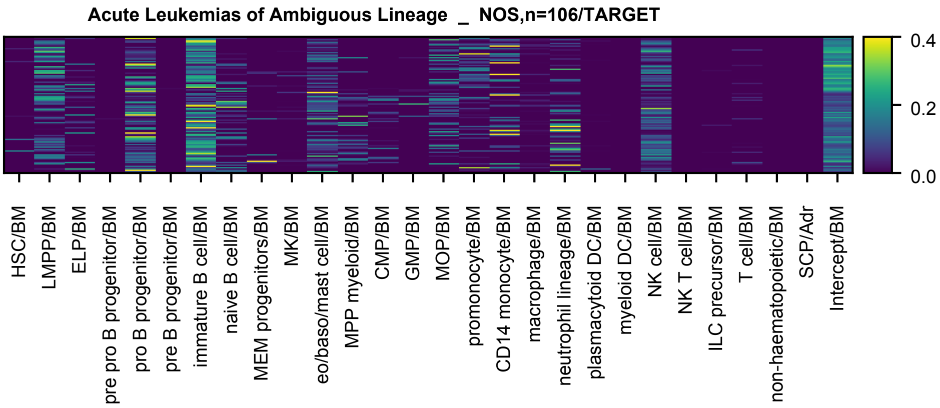
Acute Leukemias of Ambiguous Lineage _ NOS,n=106/TARGET
0.4
0.2
0.0
HSC/BM
LMPP/BM
ELP/BM
pre pro B progenitor/BM
pro B progenitor/BM
pre B progenitor/BM
immature B cell/BM
naive B cell/BM
MEM progenitors/BM
MK/BM
eo/baso/mast cell/BM
MPP myeloid/BM
CMP/BM
GMP/BM
MOP/BM
promonocyte/BM
CD14 monocyte/BM
macrophage/BM
neutrophil lineage/BM
plasmacytoid DC/BM
myeloid DC/BM
NK cell/BM
NK T cell/BM
ILC precursor/BM
T cell/BM
non-haematopoietic/BM
SCP/Adr
Intercept/BM

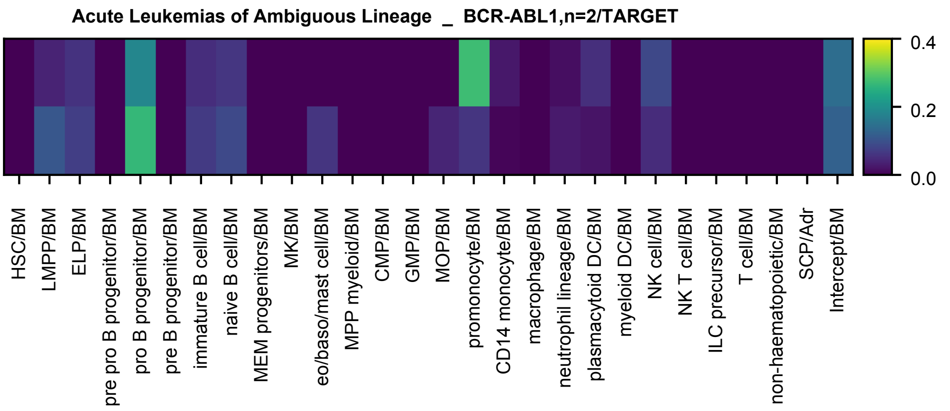
Acute Leukemias of Ambiguous Lineage _ BCR-ABL1,n=2/TARGET
0.4
0.2
0.0
HSC/BM
LMPP/BM
ELP/BM
pre pro B progenitor/BM
pro B progenitor/BM
pre B progenitor/BM
immature B cell/BM
naive B cell/BM
MEM progenitors/BM
MK/BM
eo/baso/mast cell/BM
MPP myeloid/BM
CMP/BM
GMP/BM
MOP/BM
promonocyte/BM
CD14 monocyte/BM
macrophage/BM
neutrophil lineage/BM
plasmacytoid DC/BM
myeloid DC/BM
NK cell/BM
NK T cell/BM
ILC precursor/BM
T cell/BM
non-haematopoietic/BM
SCP/Adr
Intercept/BM

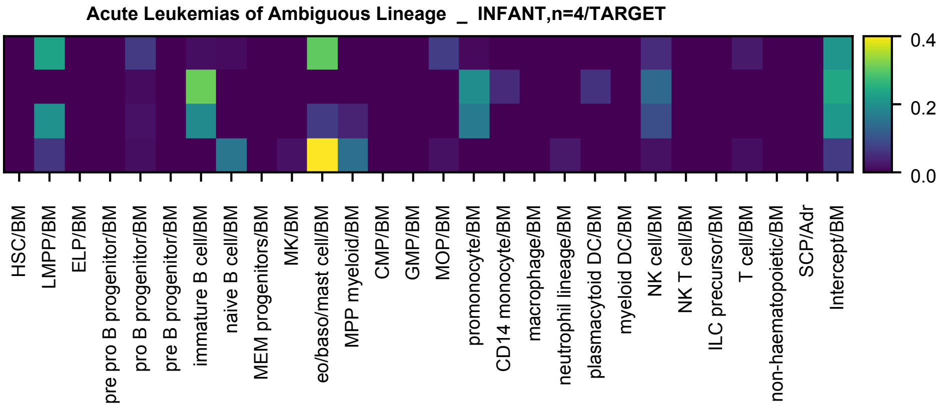
Acute Leukemias of Ambiguous Lineage _ INFANT,n=4/TARGET
0.4
0.2
0.0
HSC/BM
LMPP/BM
ELP/BM
pre pro B progenitor/BM
pro B progenitor/BM
pre B progenitor/BM
immature B cell/BM
naive B cell/BM
MEM progenitors/BM
MK/BM
eo/baso/mast cell/BM
MPP myeloid/BM
CMP/BM
GMP/BM
MOP/BM
promonocyte/BM
CD14 monocyte/BM
macrophage/BM
neutrophil lineage/BM
plasmacytoid DC/BM
myeloid DC/BM
NK cell/BM
NK T cell/BM
ILC precursor/BM
T cell/BM
non-haematopoietic/BM
SCP/Adr
Intercept/BM

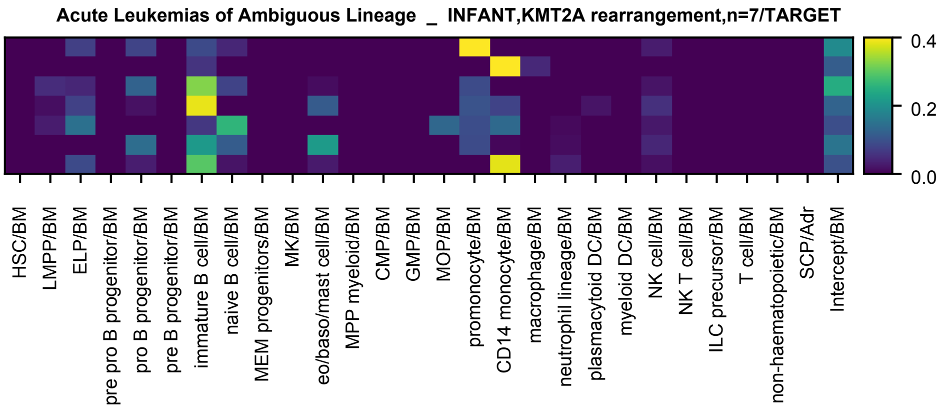
Acute Leukemias of Ambiguous Lineage _ INFANT,KMT2A rearrangement,n=7/TARGET
0.4
0.2
0.0
HSC/BM
LMPP/BM
ELP/BM
pre pro B progenitor/BM
pro B progenitor/BM
pre B progenitor/BM
immature B cell/BM
naive B cell/BM
MEM progenitors/BM
MK/BM
eo/baso/mast cell/BM
MPP myeloid/BM
CMP/BM
GMP/BM
MOP/BM
promonocyte/BM
CD14 monocyte/BM
macrophage/BM
neutrophil lineage/BM
plasmacytoid DC/BM
myeloid DC/BM
NK cell/BM
NK T cell/BM
ILC precursor/BM
T cell/BM
non-haematopoietic/BM
SCP/Adr
Intercept/BM

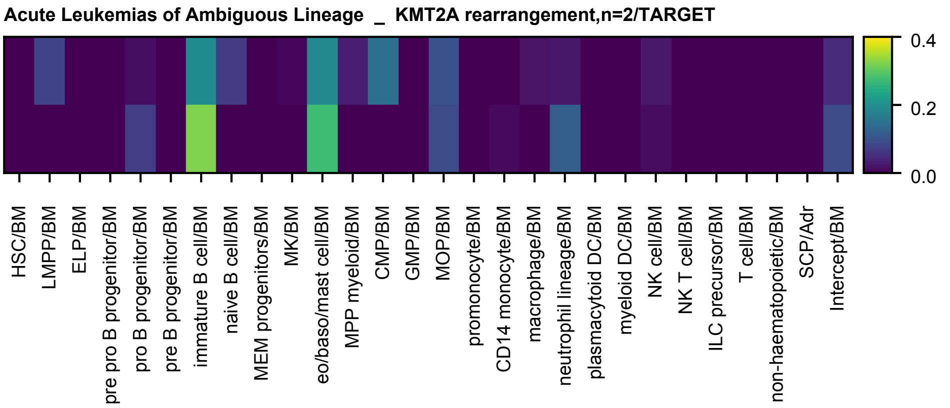
Acute Leukemias of Ambiguous Lineage _ KMT2A rearrangement,n=2/TARGET
0.4
0.2
0.0
HSC/BM
LMPP/BM
ELP/BM
pre pro B progenitor/BM
pro B progenitor/BM
pre B progenitor/BM
immature B cell/BM
naive B cell/BM
MEM progenitors/BM
MK/BM
eo/baso/mast cell/BM
MPP myeloid/BM
CMP/BM
GMP/BM
MOP/BM
promonocyte/BM
CD14 monocyte/BM
macrophage/BM
neutrophil lineage/BM
plasmacytoid DC/BM
myeloid DC/BM
NK cell/BM
NK T cell/BM
ILC precursor/BM
T cell/BM
non-haematopoietic/BM
SCP/Adr
Intercept/BM

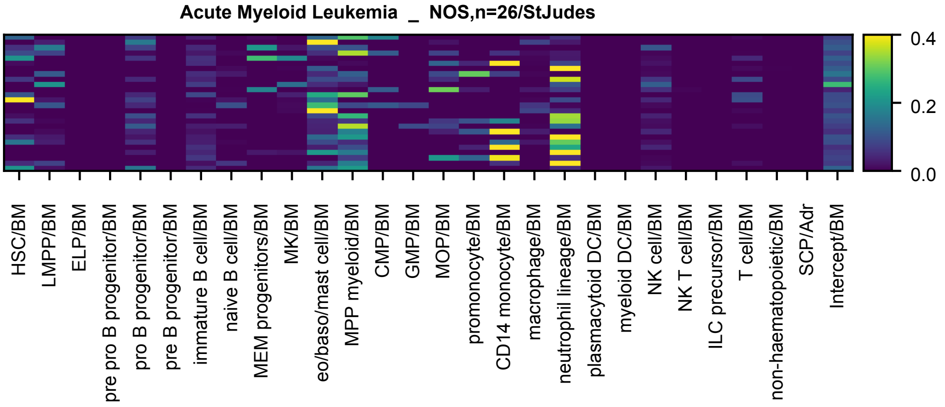
Acute Myeloid Leukemia _ NOS,n=26/StJudes
HSC/BM
LMPP/BM
ELP/BM
pre pro B progenitor/BM
pro B progenitor/BM
pre B progenitor/BM
immature B cell/BM
naive B cell/BM
MEM progenitors/BM
MK/BM
eo/baso/mast cell/BM
MPP myeloid/BM
CMP/BM
GMP/BM
MOP/BM
promonocyte/BM
CD14 monocyte/BM
macrophage/BM
neutrophil lineage/BM
plasmacytoid DC/BM
myeloid DC/BM
NK cell/BM
NK T cell/BM
ILC precursor/BM
T cell/BM
non-haematopoietic/BM
SCP/Adr
Intercept/BM
0.4
0.2
0.0

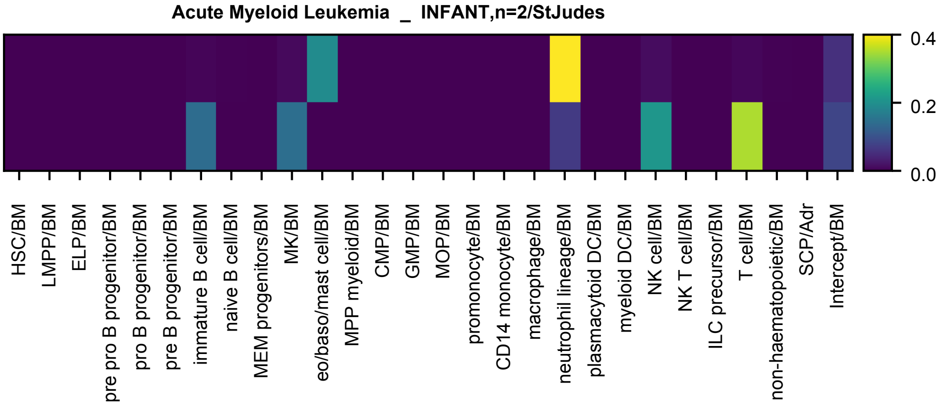
Acute Myeloid Leukemia _ INFANT,n=2/StJudes
HSC/BM
LMPP/BM
ELP/BM
pre pro B progenitor/BM
pro B progenitor/BM
pre B progenitor/BM
immature B cell/BM
naive B cell/BM
MEM progenitors/BM
MK/BM
eo/baso/mast cell/BM
MPP myeloid/BM
CMP/BM
GMP/BM
MOP/BM
promonocyte/BM
CD14 monocyte/BM
macrophage/BM
neutrophil lineage/BM
plasmacytoid DC/BM
myeloid DC/BM
NK cell/BM
NK T cell/BM
ILC precursor/BM
T cell/BM
non-haematopoietic/BM
SCP/Adr
Intercept/BM
0.4
0.2
0.0

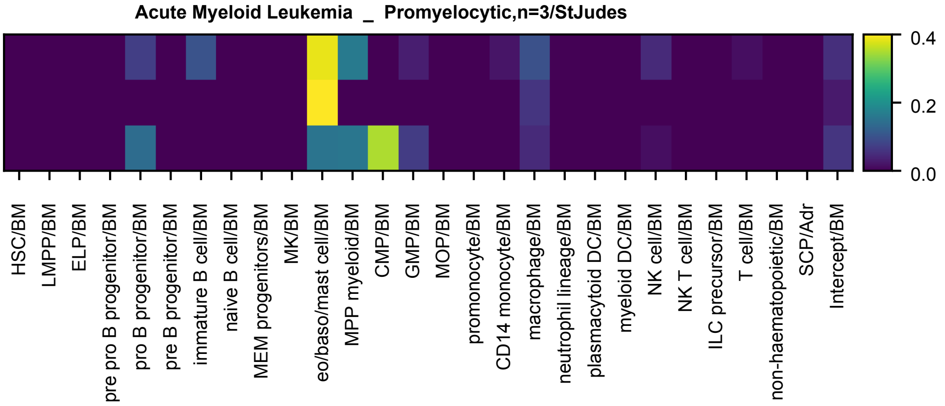
Acute Myeloid Leukemia _ Promyelocytic,n=3/StJudes
HSC/BM
LMPP/BM
ELP/BM
pre pro B progenitor/BM
pro B progenitor/BM
pre B progenitor/BM
immature B cell/BM
naive B cell/BM
MEM progenitors/BM
MK/BM
eo/baso/mast cell/BM
MPP myeloid/BM
CMP/BM
GMP/BM
MOP/BM
promonocyte/BM
CD14 monocyte/BM
macrophage/BM
neutrophil lineage/BM
plasmacytoid DC/BM
myeloid DC/BM
NK cell/BM
NK T cell/BM
ILC precursor/BM
T cell/BM
non-haematopoietic/BM
SCP/Adr
Intercept/BM
0.4
0.2
0.0

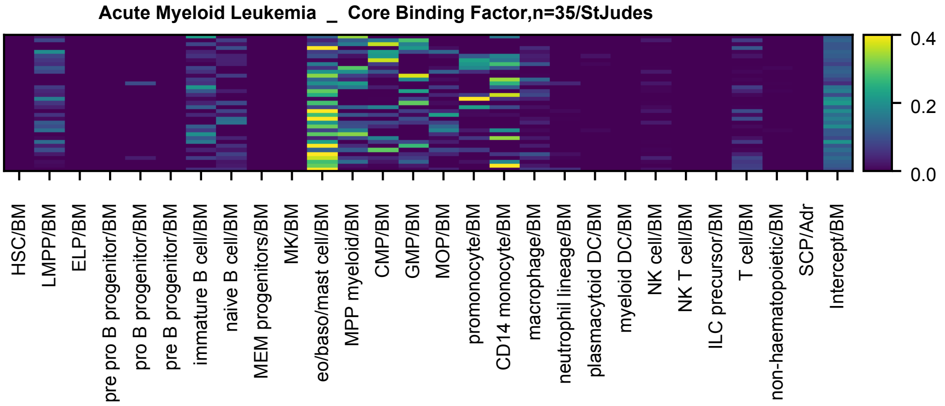
Acute Myeloid Leukemia _ Core Binding Factor,n=35/StJudes
HSC/BM
LMPP/BM
ELP/BM
pre pro B progenitor/BM
pro B progenitor/BM
pre B progenitor/BM
immature B cell/BM
naive B cell/BM
MEM progenitors/BM
MK/BM
eo/baso/mast cell/BM
MPP myeloid/BM
CMP/BM
GMP/BM
MOP/BM
promonocyte/BM
CD14 monocyte/BM
macrophage/BM
neutrophil lineage/BM
plasmacytoid DC/BM
myeloid DC/BM
NK cell/BM
NK T cell/BM
ILC precursor/BM
T cell/BM
non-haematopoietic/BM
SCP/Adr
Intercept/BM
0.4
0.2
0.0

Acute Myeloid Leukemia _ KMT2A rearrangement,n=9/StJudes

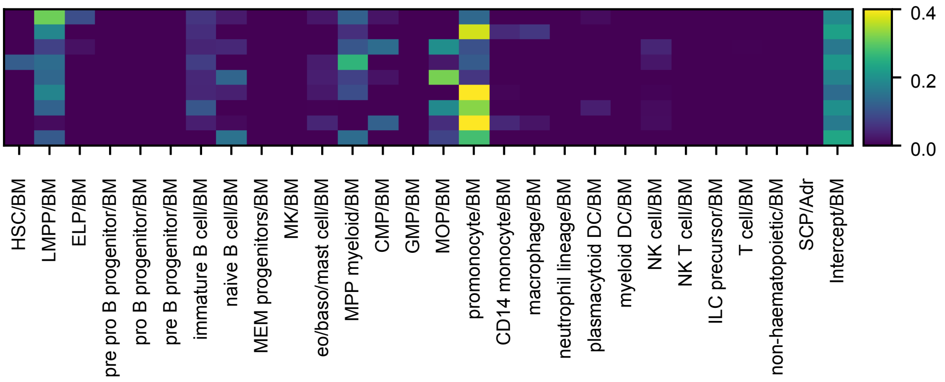


Acute Myeloid Leukemia _ NOS,n=476/TARGET

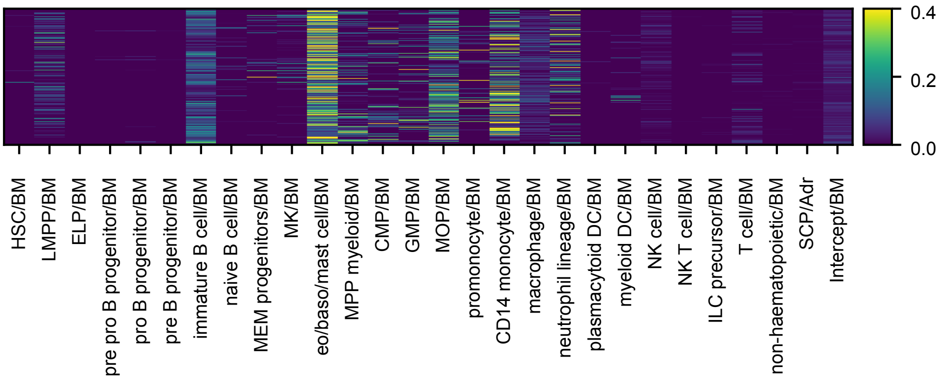


Acute Myeloid Leukemia _ INFANT,n=21/TARGET

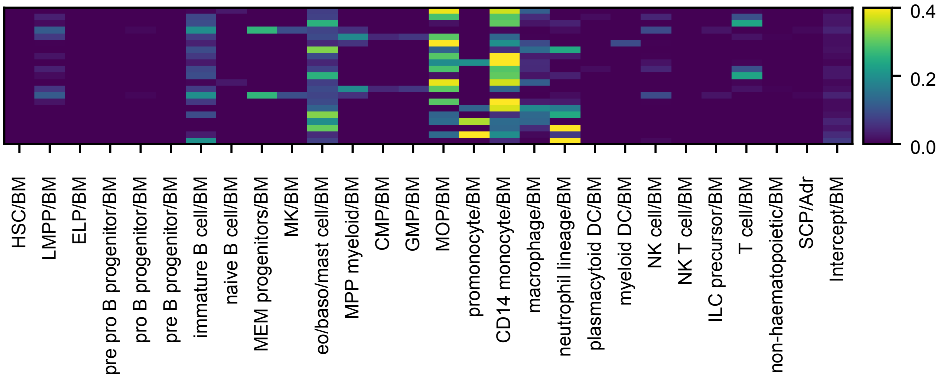


Acute Myeloid Leukemia _ INFANT,KMT2A rearrangement,n=17/TARGET

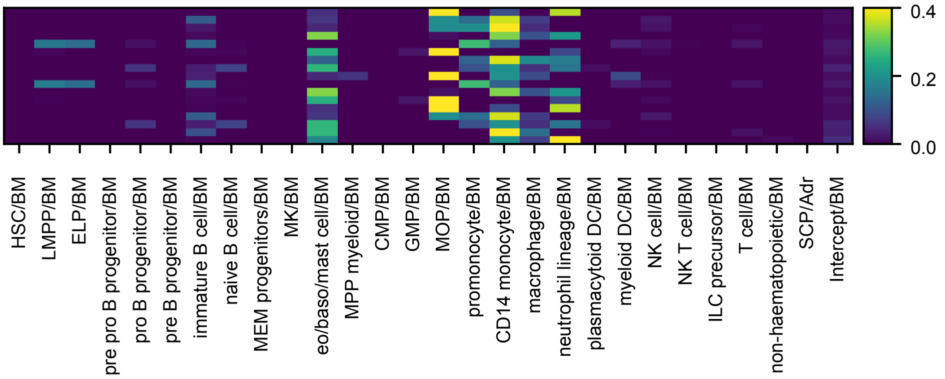

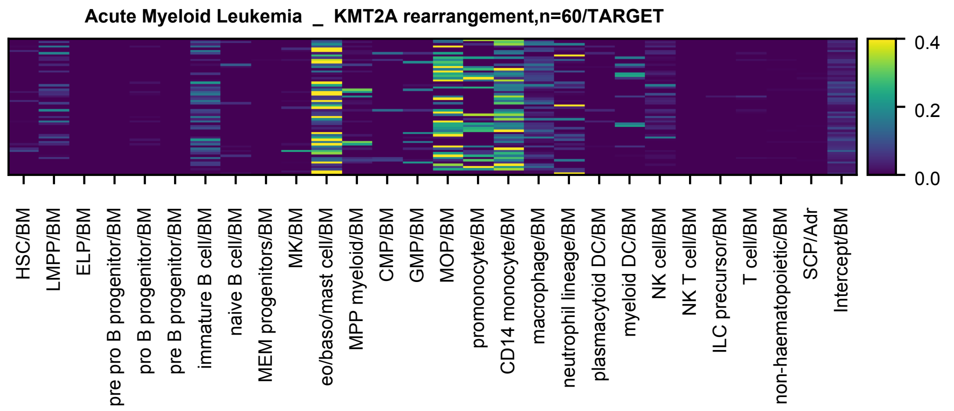
Acute Myeloid Leukemia _ KMT2A rearrangement,n=60/TARGET
0.4
0.2
0.0
HSC/BM
LMPP/BM
ELP/BM
pre pro B progenitor/BM
pro B progenitor/BM
pre B progenitor/BM
immature B cell/BM
naive B cell/BM
MEM progenitors/BM
MK/BM
eo/baso/mast cell/BM
MPP myeloid/BM
CMP/BM
GMP/BM
MOP/BM
promonocyte/BM
CD14 monocyte/BM
macrophage/BM
neutrophil lineage/BM
plasmacytoid DC/BM
myeloid DC/BM
NK cell/BM
NK T cell/BM
ILC precursor/BM
T cell/BM
non-haematopoietic/BM
SCP/Adr
Intercept/BM

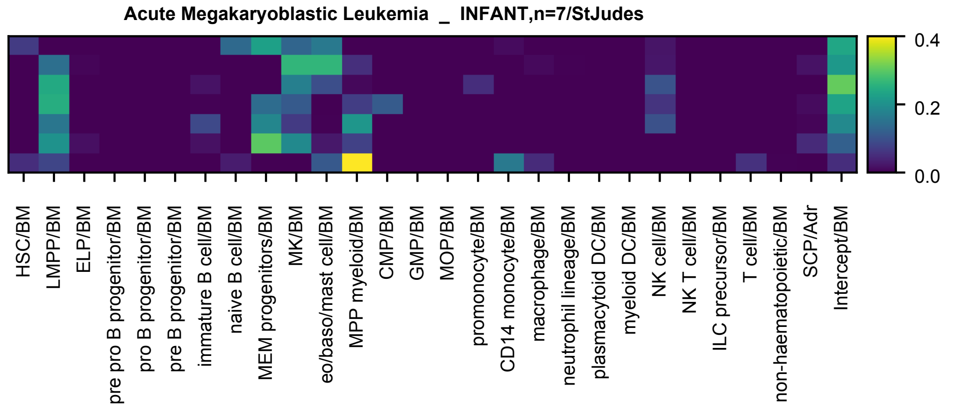
Acute Megakaryoblastic Leukemia _ INFANT,n=7/StJudes
0.4
0.2
0.0
HSC/BM
LMPP/BM
ELP/BM
pre pro B progenitor/BM
pro B progenitor/BM
pre B progenitor/BM
immature B cell/BM
naive B cell/BM
MEM progenitors/BM
MK/BM
eo/baso/mast cell/BM
MPP myeloid/BM
CMP/BM
GMP/BM
MOP/BM
promonocyte/BM
CD14 monocyte/BM
macrophage/BM
neutrophil lineage/BM
plasmacytoid DC/BM
myeloid DC/BM
NK cell/BM
NK T cell/BM
ILC precursor/BM
T cell/BM
non-haematopoietic/BM
SCP/Adr
Intercept/BM

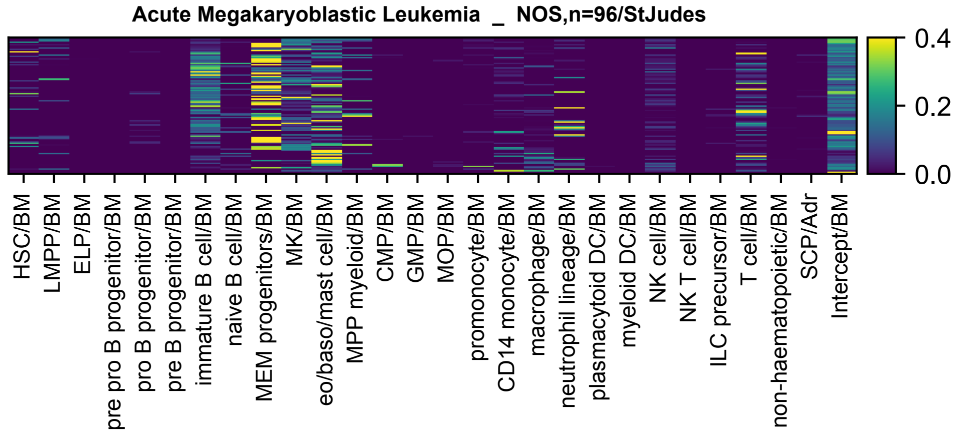
Acute Megakaryoblastic Leukemia _ NOS,n=96/StJudes
0.4
0.2
0.0
HSC/BM
LMPP/BM
ELP/BM
pre pro B progenitor/BM
pro B progenitor/BM
pre B progenitor/BM
immature B cell/BM
naive B cell/BM
MEM progenitors/BM
MK/BM
eo/baso/mast cell/BM
MPP myeloid/BM
CMP/BM
GMP/BM
MOP/BM
promonocyte/BM
CD14 monocyte/BM
macrophage/BM
neutrophil lineage/BM
plasmacytoid DC/BM
myeloid DC/BM
NK cell/BM
NK T cell/BM
ILC precursor/BM
T cell/BM
non-haematopoietic/BM
SCP/Adr
Intercept/BM

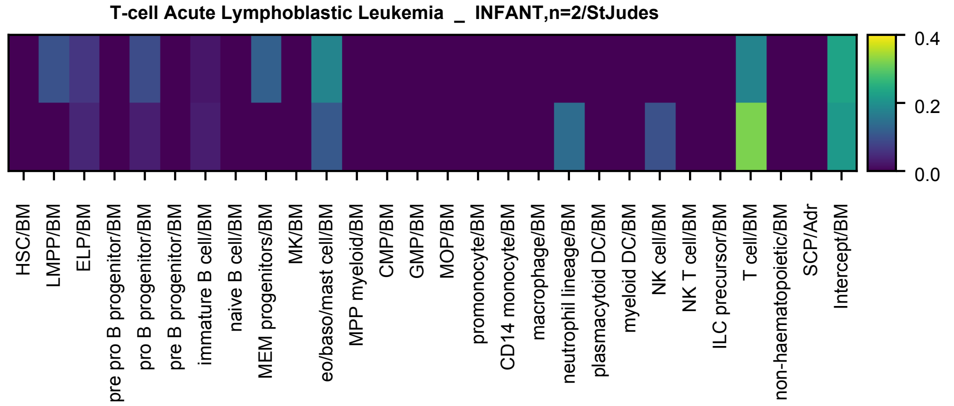
T-cell Acute Lymphoblastic Leukemia _ INFANT,n=2/StJudes
0.4
0.2
0.0
HSC/BM
LMPP/BM
ELP/BM
pre pro B progenitor/BM
pro B progenitor/BM
pre B progenitor/BM
immature B cell/BM
naive B cell/BM
MEM progenitors/BM
MK/BM
eo/baso/mast cell/BM
MPP myeloid/BM
CMP/BM
GMP/BM
MOP/BM
promonocyte/BM
CD14 monocyte/BM
macrophage/BM
neutrophil lineage/BM
plasmacytoid DC/BM
myeloid DC/BM
NK cell/BM
NK T cell/BM
ILC precursor/BM
T cell/BM
non-haematopoietic/BM
SCP/Adr
Intercept/BM

**T-cell Acute Lymphoblastic Leukemia _ KMT2A rearrangement,n=2/StJudes**

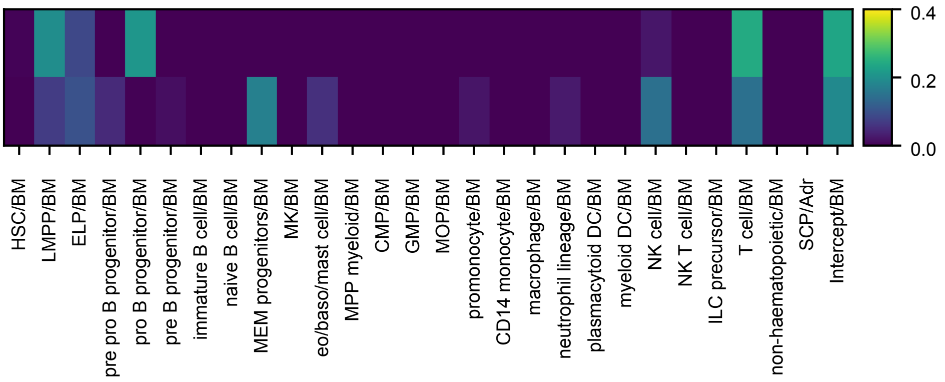


**T-cell Acute Lymphoblastic Leukemia _ NOS,n=15/StJudes**

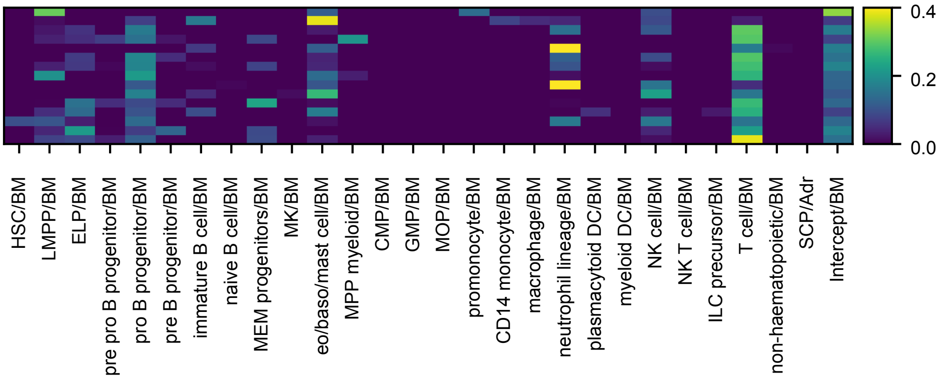


**T-cell Acute Lymphoblastic Leukemia _ NOS,n=265/TARGET**

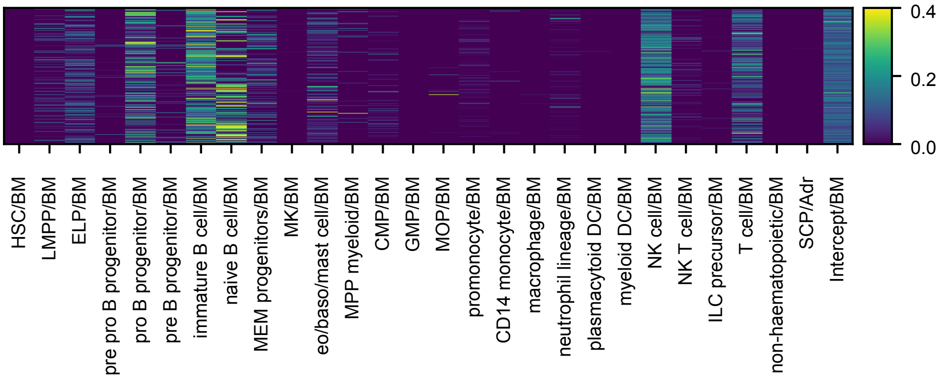


**SCP _ SCP**

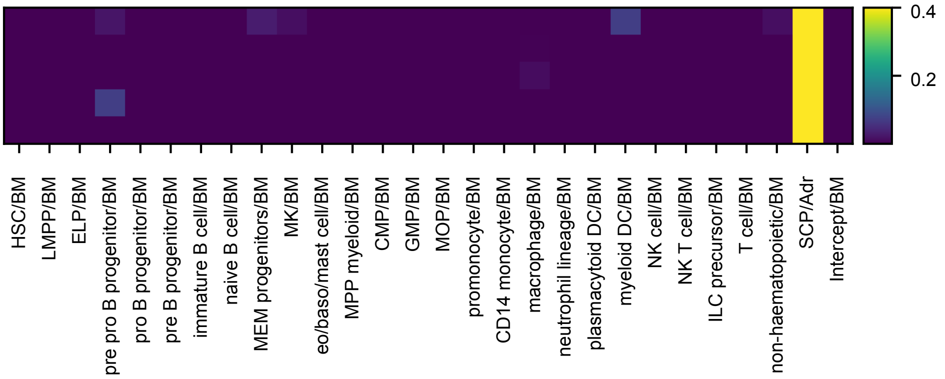

## Supplementary Table legends

**Supplementary Table 1.** Categories of childhood leukemia from St Jude and TARGET cohorts

**Supplementary Table 2.** Mapping of fetal bone marrow reference populations to detailed cell_id in fetal bone marrow dataset

**Supplementary Table 3.** Overview of study cohort.
Case IDs indicate how samples are referenced in manuscript, figures and supplementary materials. PR, PD, and Sc IDs are Sanger Institute references for bulk RNA-seq, DNA-seq and single-cell RNA-seq data.

**Supplementary Table 4.** Immunophenotypes of infant leukemia samples.
Diagnostic immunophenotypes of leukemia samples. Antigen selection and reporting was performed according to local clinical practice. NT= not tested. Numerical data indicates % of leukemia cells expressing given antigen. + indicates expressed by leukaemia cells; - indicates not expressed and +/- indicates expressed by a proportion of leukemia cells. Row colors denote immunophenotype genes shown in **Extended Data Figure 3**.

**Supplementary Table 5.** Differentially expressed genes in infant leukemia subtypes.
Differentially expressed genes between *NUTM1* infant B-ALL K*MT2A*-rearranged infant B-ALL from bulk and single-cell mRNA data

**Supplementary Table 6**. List of single nucleotide variants in lineage-switch case analysis

**Supplementary Table 7**. Gene ontology annotation of differentially expressed genes between infant ALL and ELP cells from bulk and single cell mRNA data, including list of surface markers.

**Supplementary Table 8**. Differentially expressed genes between *KMT2A*-rearranged infant AML and monocyte progenitor (MOP) cells single-cell mRNA data.
